# 2D materials: increscent quantum flatland with immense potential for applications

**DOI:** 10.1186/s40580-022-00317-7

**Published:** 2022-06-06

**Authors:** Pranay Ranjan, Snehraj Gaur, Himanshu Yadav, Ajay B. Urgunde, Vikas Singh, Avit Patel, Kusum Vishwakarma, Deepak Kalirawana, Ritu Gupta, Prashant Kumar

**Affiliations:** 1 Department of Metallurgical and Materials Engineering, Indian Institute of Technology Jodhpur, Karwar, 342037 Rajasthan India; 2 Advanced Materials and Devices Laboratory, Department of Chemistry, Indian Institute of Technology Jodhpur, Karwar, 342037 Rajasthan India; 3 Global Innovative Centre for Advanced Nanomaterials (GICAN), College of Engineering, Science and Environment (CESE), School of Engineering, The University of Newcastle, University Drive, Callaghan, NSW 2308 Australia

**Keywords:** 2D materials, Synthesis, Characterization, Applications

## Abstract

Quantum flatland i.e., the family of two dimensional (2D) quantum materials has become increscent and has already encompassed elemental atomic sheets (Xenes), 2D transition metal dichalcogenides (TMDCs), 2D metal nitrides/carbides/carbonitrides (MXenes), 2D metal oxides, 2D metal phosphides, 2D metal halides, 2D mixed oxides, etc. and still new members are being explored. Owing to the occurrence of various structural phases of each 2D material and each exhibiting a unique electronic structure; bestows distinct physical and chemical properties. In the early years, world record electronic mobility and fractional quantum Hall effect of graphene attracted attention. Thanks to excellent electronic mobility, and extreme sensitivity of their electronic structures towards the adjacent environment, 2D materials have been employed as various ultrafast precision sensors such as gas/fire/light/strain sensors and in trace-level molecular detectors and disease diagnosis. 2D materials, their doped versions, and their hetero layers and hybrids have been successfully employed in electronic/photonic/optoelectronic/spintronic and straintronic chips. In recent times, quantum behavior such as the existence of a superconducting phase in moiré hetero layers, the feasibility of hyperbolic photonic metamaterials, mechanical metamaterials with negative Poisson ratio, and potential usage in second/third harmonic generation and electromagnetic shields, etc. have raised the expectations further. High surface area, excellent young’s moduli, and anchoring/coupling capability bolster hopes for their usage as nanofillers in polymers, glass, and soft metals. Even though lab-scale demonstrations have been showcased, large-scale applications such as solar cells, LEDs, flat panel displays, hybrid energy storage, catalysis (including water splitting and CO_2_ reduction), etc. will catch up. While new members of the flatland family will be invented, new methods of large-scale synthesis of defect-free crystals will be explored and novel applications will emerge, it is expected. Achieving a high level of in-plane doping in 2D materials without adding defects is a challenge to work on. Development of understanding of inter-layer coupling and its effects on electron injection/excited state electron transfer at the 2D-2D interfaces will lead to future generation heterolayer devices and sensors.

## Introduction to 2D materials

**Fig. 1.**
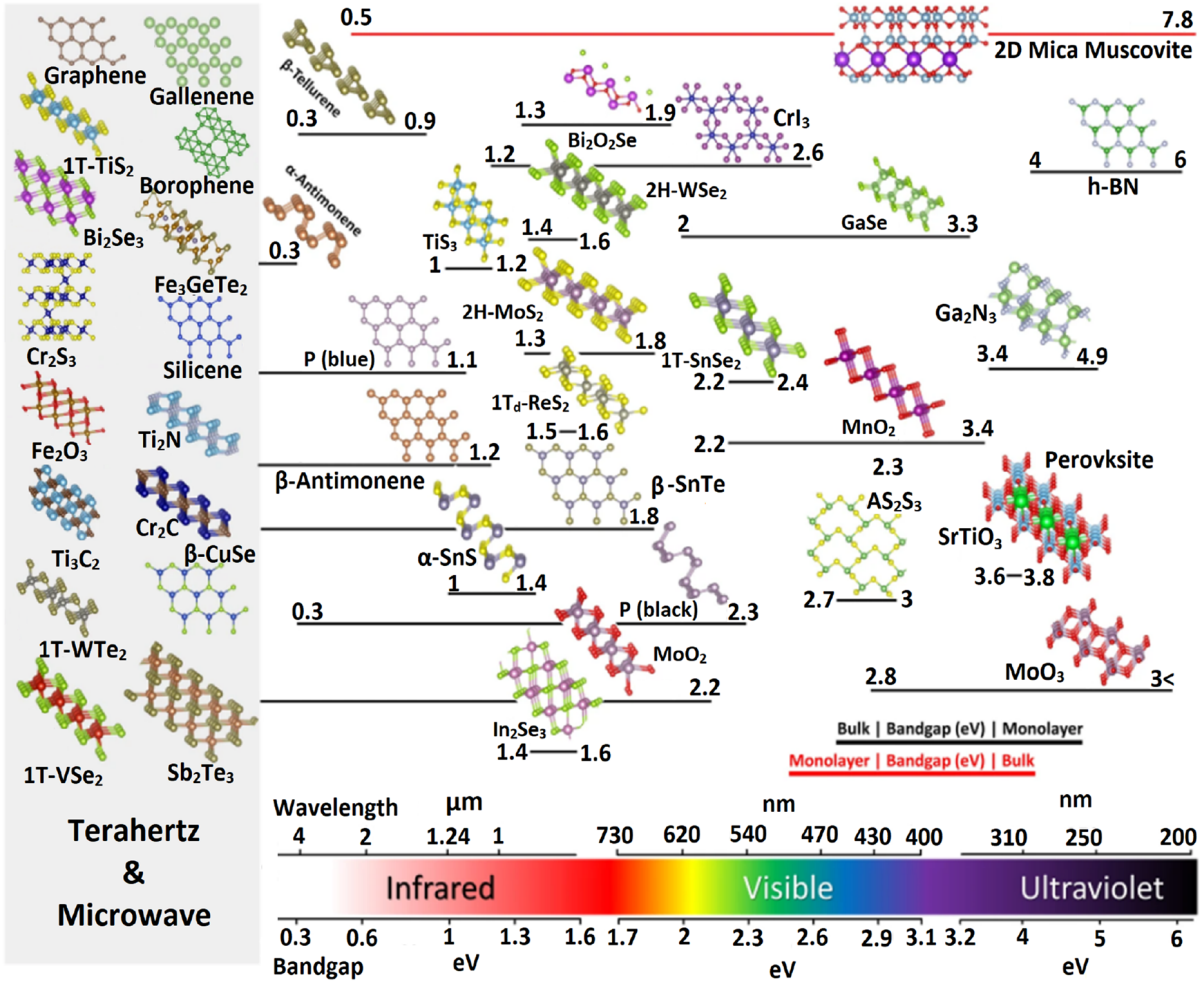
2D materials are chosen for their experimental significance and demonstration, with depictions of a perspective view of their crystal structures. The arrangement is following their bandgap, guided by the bottom wavelength/bandgap scale, whereas the bar beneath each structure indicates the bandgap range from bulk to monolayer. Typically, the bulk bandgap is smaller than that of its monolayer (black bars), but there are exceptions (red bars). 2D materials on the far left, indicated by a gray box, are zero or near-zero bandgap, metallic, or semimetallic [8]

Pioneering breakthrough by Geim and Novosolev on micromechanical exfoliation to obtain sp^2^ bonded atomic sheet of carbon i.e., *graphene* from naturally occurring layered material graphite in 2004 led to the 2010 Nobel prize in Physics [1]. Graphene on investigation exhibited a Dirac nature and the quantum Hall effect. Apart from the single-crystal nature at atomic thickness, several attributes such as high surface area, world record electronic mobility, excellent thermal conductivity, superior Young's modulus, and chemical stability up to 300 °C were remarkable. Graphene has revolutionized materials science and engineering. Applications spanning over electronic chips, electron tunneling devices, LEDs, solar cells, energy sector, laser shields, light combat aircraft, night vision cameras, advanced electronic gadgets, SERS based molecular detection, gas sensing prompt disease (viral/cancer/diabetic) diagnosis [2–19], etc. have rendered it a wonder material. Moreover, Moiré-hetero layers of graphene have been amusing with interesting quantum behavior in terms of its unconventional superconducting and magnetic behavior. Quantum electronic signatures emerging out of graphene has established it as one of the best-known quantum materials and has opened up enormous possibilities [20]. Due to the lack of sufficient charge carriers, and zero bandgap, in graphene the ON/OFF ratio exhibited was found to be negligible, and this hampers its prospect as an ideal candidate active layer for electronic chips. To overcome these limitations, doping of graphene lattice [21–23] and its hybridization [24] with various two/three-dimensional materials have been devised. However, parallelly, new 2D materials such as elemental atomic sheets also called Xenes such as borophene, phosphorene, Stannene, Germanene, etc. were developed in the following years [25, 26]. Parallelly, boron nitride (BN) and TMDCs (e.g., Molybdenum disulfide, Tungsten disulfide) were developed [27]. Apart from TMDCs, 2D versions of metal oxides [28, 29] and metal phosphides [30] were discovered, while MXenes (Metal nitrides, metal carbides and carbonitrides, etc.) developed thereafter [31, 32]. A calendar of the 2D materials family is exhibited in Fig. 1. In this article, we elaborate on the evolution of the family of 2D materials, its synthesis protocols, and various technological applications. Challenges being faced by the quantum flatland have been elaborated. Furthermore, the scope and role of 2D materials in upcoming technologies have been presented.

## Synthesis of 2D materials

**Fig. 2:**
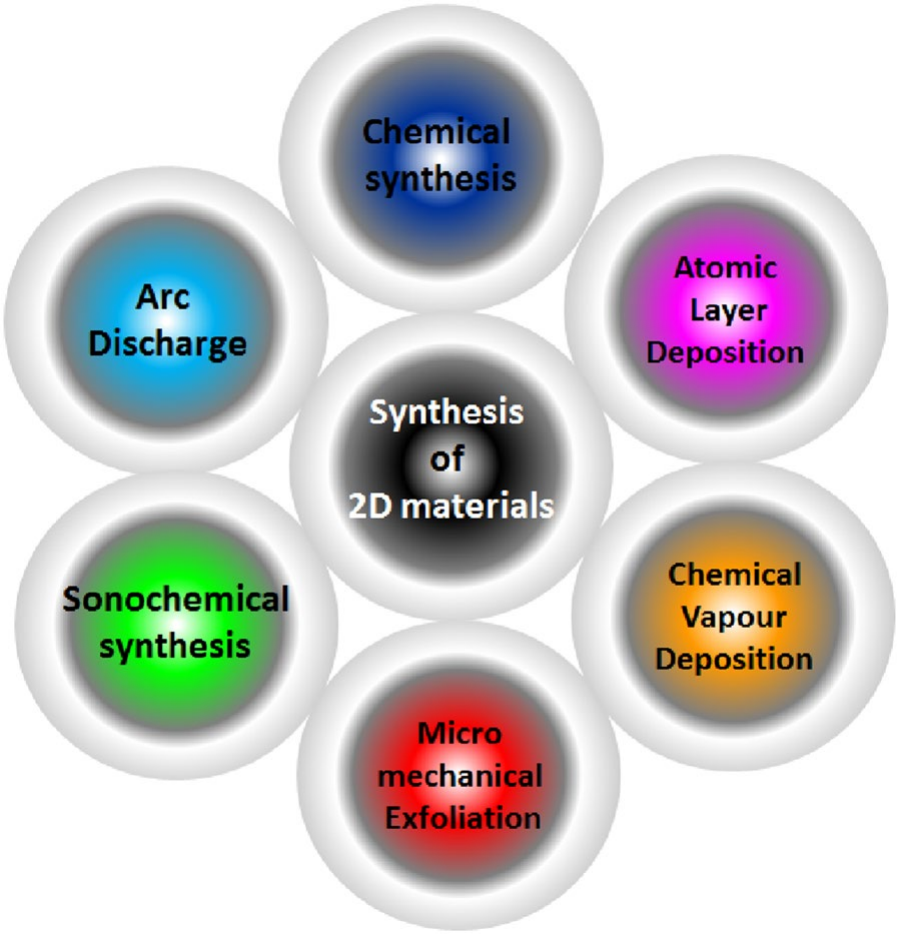
Methods of synthesis of 2D materials

Synthesis methods for graphene include sonochemical exfoliation [2, 33, 34], arc discharge technique [35], Photo exfoliation [36, 37], Chemical vapor deposition [38], Laser chemical vapor deposition [39], Microwave plasma chemical vapor deposition [40] and unzipping of carbon nanotubes [41–43]. Chemical vapor deposition and arc discharge techniques are widely used for electronic devices due to their control over defect and thickness and thus helps in delivering the high-end product. However, these techniques are not suitable for industrial-scale due to the involvement of toxic gases, cumbersome deposition techniques, and the need for skilled labor at every stage of growth. The cost of the end product is generally very high and thus limits the use of this technique to limited areas such as use in defense products, etc. To address the scalability issues, modified Hummer’s method of oxidative exfoliation [44] followed by reduction has been employed widely by the scientific community. All these chemical-based intercalation methods are the fundamental techniques for large-scale production and involve interaction between solute and the material as well as an additional parameter such as the ultrasonic wave frequency/exothermic energy/photon energy or a blend of the two. The main advantage of using these techniques is that they can make 2D materials at a large scale at a low cost and don’t involve the use of any toxic gases. However, these techniques involve certain issues like the inclusion of defects, functionalization, crumplings, wrinkles, fragmentation, and non-uniform sheet sizes and thus limit the use in electronic devices (particularly due to the opening of the bandgap). In addition, to remove the functionalities and end up with a clean pristine sheet different methodologies are being introduced. Among oxygen functionality reduction strategies, annealing in a hot air oven, microwave annealing [45], and laser reduction [46–48] are a few. Methods of synthesis for graphene have been widely adopted for the synthesis of other 2D materials too. For example, Ranjan et al. adopted micromechanical exfoliation and modified Hummer’s method for the synthesis of borophene [49, 50] and micromechanical exfoliation. Sahu et al. [51] recently carried out a modified Hummer method of oxidative exfoliation of MoS_2_ and BN [52]. Most of the synthesis techniques for graphene are material independent and can conveniently be employed for various Xenes and other 2D materials too. Methods of synthesis of 2D materials have been summarized in Fig. 2.

## Characterization of 2D materials

Graphene, the atomic thin layer of carbon atom arranged in a hexagonal geometry contains sp^2^ hybridized carbon atoms. It has sp^2^-hybridized planar bonds making 120° with each other, which gives the hexagonal arrangement of carbon atoms. Carbon has four valence electrons, out of which three electrons get hybridized in a sp^2^ configuration (thus forming a bond), and the fourth electron forms the half-filled 2p_z_, thus leading to the formation of π covalent bonds. This σ bond is considered to be the reason behind the unprecedented chemical and mechanical properties of graphene concerning other 2D materials. However, the electronic properties are related to the p-orbital that is always perpendicular to the lattice. The p-orbital usually leads to the formation of a covalent bond and forms the electronic π band. These half-filled bands result in exceptionally high electronic mobility or the carrier transport of graphene. Contrary to this the chemist approach suggests that the key to enhanced electronic property of graphene lies in the orthogonality and non-interaction of the π and π* states respectively. It has also been an area of debate that the linear dispersion observed in the graphene monolayer sheets is also due to the two orthogonal states. Thus, a chemical approach suggests that the structure and the nature of the bond (covalent) both lead to high mechanical strength and a higher value of the optical phonon frequency. These significantly high values of the phonon frequency (1600 cm^−1^) in graphene as compared to the silicon (~ 520 cm^−1^), III–V compounds (~ 310 cm^−1^) lead to less optical phonon scattering than in comparison to the other existing conventional semiconductors. In addition, low scattering of the phonons and zero mass, as well as significantly high values of the Fermi velocity carriers, induces ballistic regime and quantum tunneling which otherwise is not possible with other conventional semiconductors [1–4]. However, the experimentally observed values of the mobility in the graphene are much lower than the theoretically predicted values due to lattice defects, edge effect, ripples, crumples, or the presence of ad-atoms [4–12]. In addition, the electronic properties of the 2D materials further rely on the atomic number (electrons available), size of the atoms, chemical bonding, crystallography structure, and strain in the system.

**Fig. 3:**
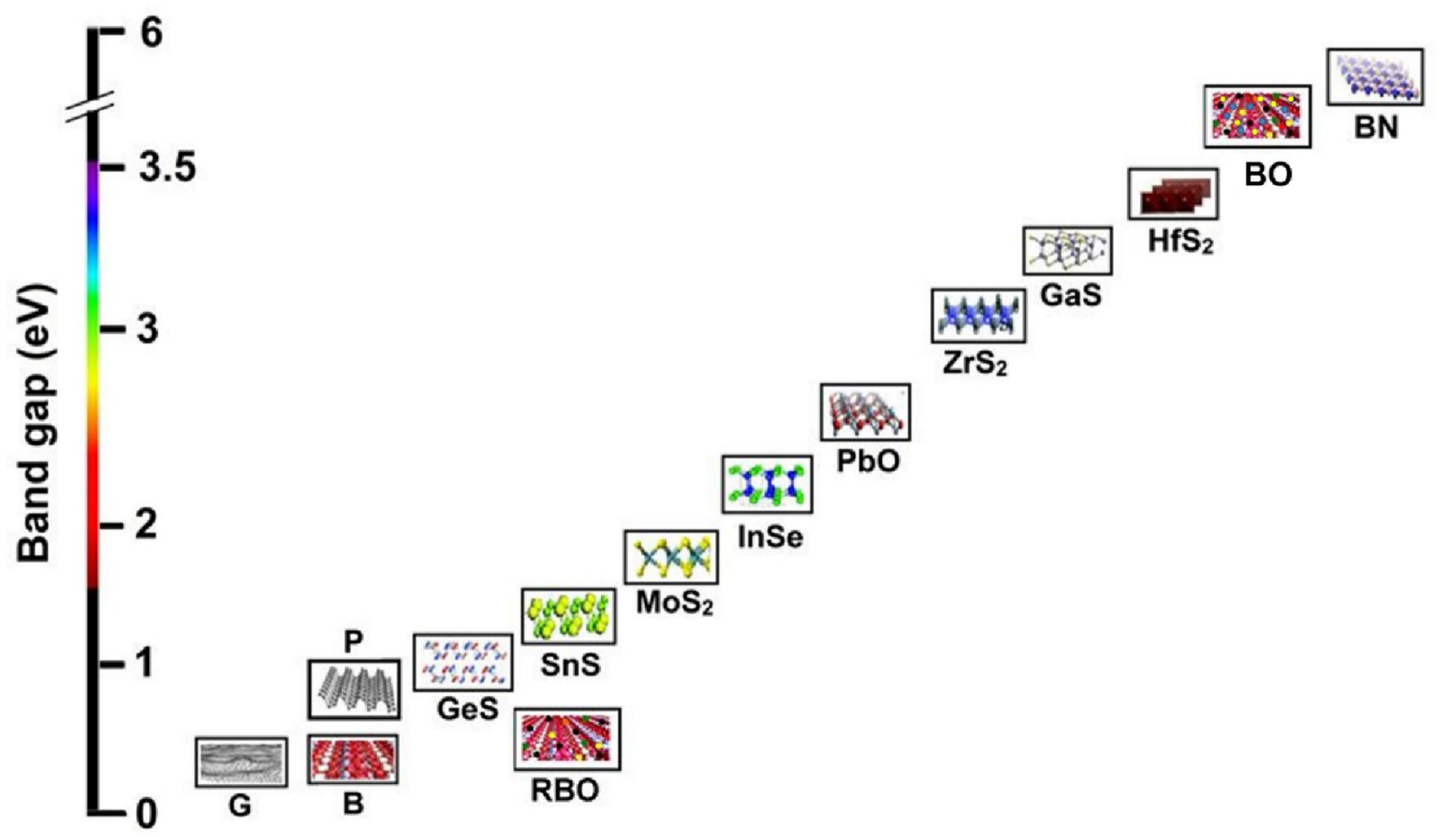
Band gaps chart for the family of 2D materials [50]

**Fig. 4:**
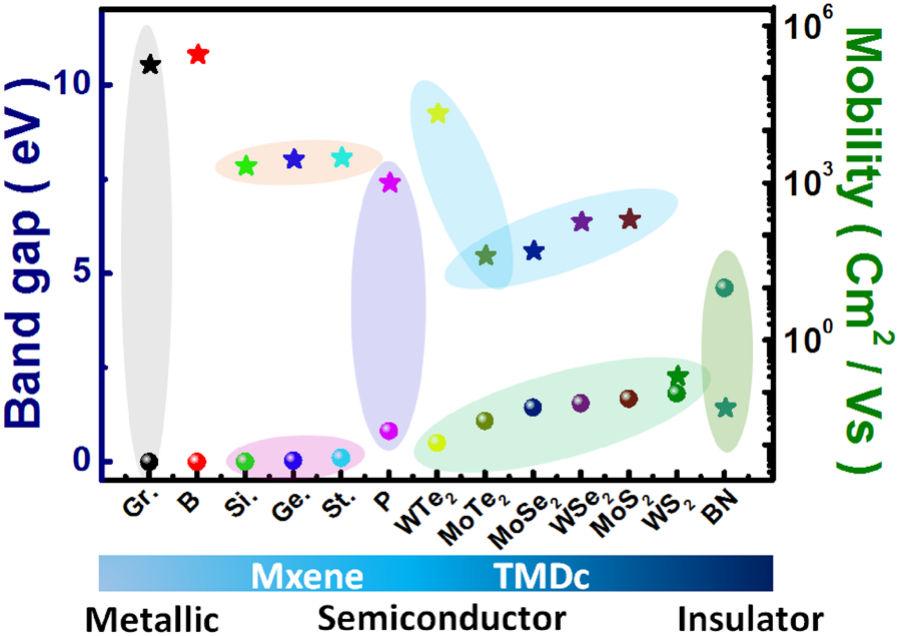
Bandgap versus mobility of various 2D materials [50]

The electronic mobility of the graphene sheets was reported to be 180,000 cm^2^/Vs, which is deemed to be the highest in comparison to atomic sheets of phosphorene, boron nitride, TMDCs, complex 2D materials such as plumbene, hematene, etc. It would be worth mentioning that the 2D material family has gone far beyond graphene to mono-elemental sheets and complex 2D oxides (as shown in Fig. 3) and is now an extremely versatile system consists metallic, semi-metallic, semiconducting, topological-insulators, and superconductors. Some of the cousins of graphene such as silicene, germanene, stannene, phosphorene, semiconducting molybdenum diselenide, tungsten diselenide, molybdenum disulfide, tungsten disulfide have mobilities such as 2100 cm^2^/Vs, 2800 cm^2^/Vs, 3000 cm^2^/Vs, 1000 cm^2^/Vs, 50 cm^2^/Vs, 180 cm^2^/Vs, 200 cm^2^/Vs, 0.2 cm^2^/Vs respectively (see Fig. 4).

Although these 2D materials have unique and unprecedented physical and chemical properties, they need to be synthesized in a pristine state and thus characterization techniques play the most important role in the investigation of these 2D materials' unique signatures. Among primary characterization techniques for 2D materials are microscopies (Optical, FESEM, AFM, and TEM/HRTEM) and spectroscopies (Raman, XPS, and FTIR). Most of the 2D materials have sharp characteristic Raman peaks. Graphene, for example, exhibits D, G, and 2D peaks at 1360 cm^−1^, 1560 cm^−1^, and ∼ 3250 cm^−1^ [53] with a typical I_2D_/I_G_ ratio is the number of layer sensitive and is > 3 for monolayers if it is chemically pure. BN exhibits Raman peak at ~ 1360 cm^−1^ [54]. MoS_2_ exhibits E_2g_ and A_1g_ peaks at ~ 384 and ~ 403 cm^−1^. Δ for the two Raman peaks for MoS_2_ is layer dependent. Semiconducting 2D materials are characterized by their band gaps. Tauc plot obtained from UV–Vis’s spectroscopy directly provides an estimate of the bandgap. The electronic bandgap can be obtained from R vs T measurement using the formula R = R_0_ exp. (− E_g_/k_B_T). Hall effects have widely been used for obtaining carrier concentration. Photoconductivity measurements provide information on applicability as active material in solar cells. Photoluminescence measurements are used to explore the feasibility of application for LEDs. FET device measurements yields electronic mobility and ON/OFF ratio to gauge the potential application in electronic chips. Nanomechanical testing has widely been used for evaluating young’s moduli to explore mechanical applications. Electrochemical measurements provide a hint whether a particular 2D material is suitable for energy applications (battery and supercapacitor) or not.

## Salient applications of 2D materials

### Energy storage devices

**Fig. 5:**
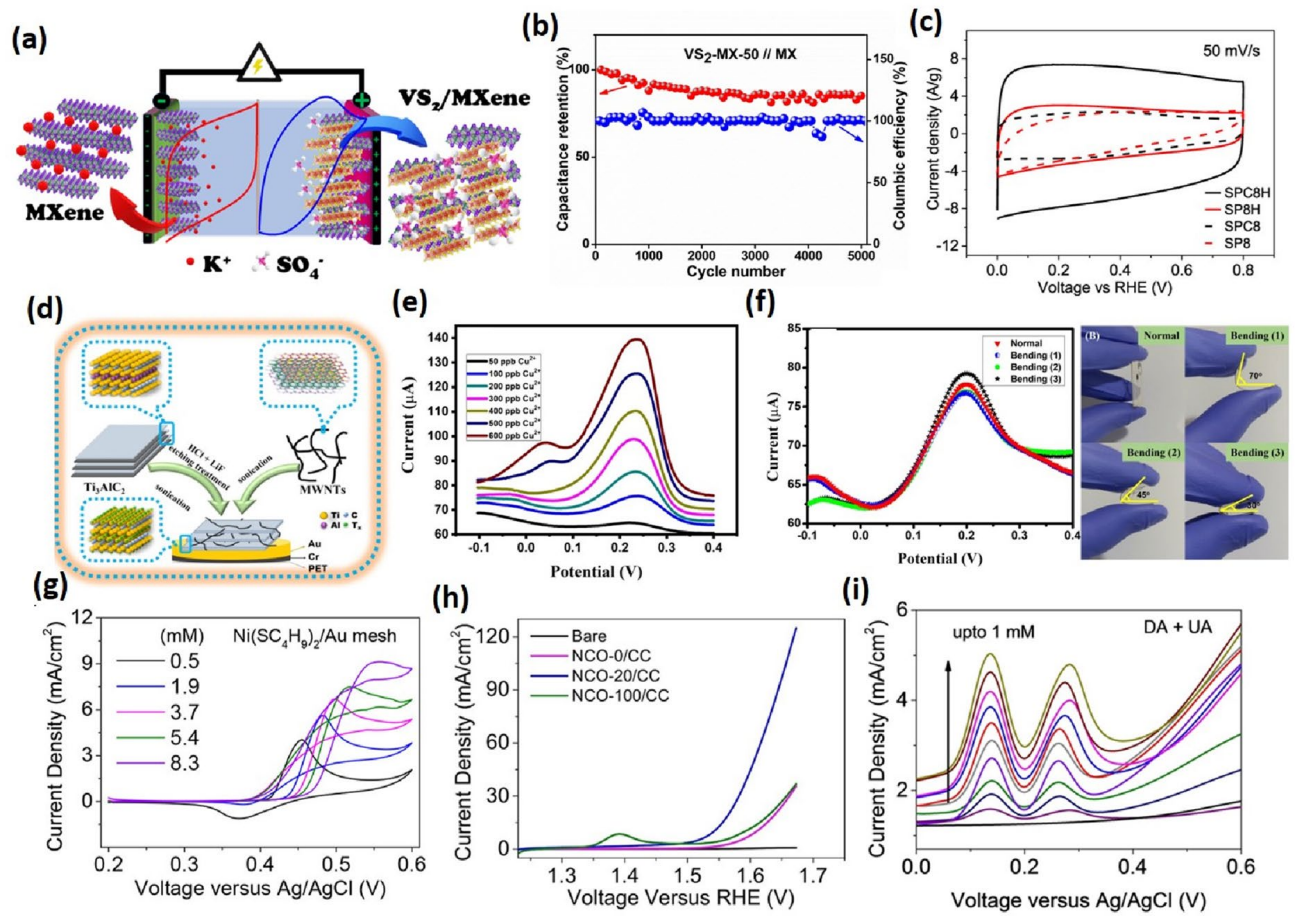
**a** Schematic representation of charge transfer and storage mechanism of the VS_2_-MX-50//MXene asymmetric device **b** Cyclic Stability (85%) and Coulombic Efficiency (∼ 100%) of the VS_2_-MX-50//MX asymmetric device at a current density of 30 A/g. Figure adapted from reference [66] **c** Cyclic Voltammograms at a scan rate of 50 mV/s in the graphitic carbon electrodes. Figure adapted from reference [76] **d** Modification process of the flexible Ti3C2Tx/MWNTs/Au/PET working electrode. **e** SWASV responses of the Ti3C2Tx/MWNTs/Au/PET electrode for the determination of Cu(II) with various high concentrations in ABS (0.1 M, pH 4.6): 50 − 600 ppb. **f** SWASV responses of the Ti3C2Tx/MWNTs/Au/PET electrode for the determination of 300 ppb Cu(II) in normal and different bending states and display of different bending processes of the fabricated electrode. Figure adapted from ref. [84] (**g**) Cyclic Voltammogram of Ni-C4SH/Au mesh as working electrodes in the glucose of different concentrations at a scan rate of 50 mV s^−^1 Figure adapted from ref [85] (**h**) LSV curves at 5 mV/s of Ni-Co oxide nanoplate Figure adapted from ref. [86] (**i**) Simultaneous measurements with increasing concentration of equimolar Dopamine and Uric Acid from 1 nM to 1000 μM, respectively (Figure adapted from ref [87])

The global rise in energy demands has paved the way for efficient energy storage devices. 2D materials with large specific surface area, high conductivity, and high electrochemical activity are promising for supercapacitors and batteries. The atomic-level thickness and high flexibility make them suitable for wearable energy storage applications [55]. Most conventional materials can’t utilize their whole structure for electrolyte ion diffusion, hindering charge storage capability. Supercapacitors store energy in the form of an electric double layer or redox reaction at the electrode surface and thus require electrode materials with high specific surface area for enhanced device performance. Numerous 2D materials for supercapacitors include graphene, TMDs, and BP for higher conductivity, MXenes, Metal oxides, and layered double hydroxides for higher electrochemical redox activity while metal–organic frameworks and covalent organic frameworks with high surface area [56, 57]. Pristine 2D materials are not adequate for direct supercapacitor fabrication due to poor structural and chemical stability in the electrochemical process, inert surface, lower electrical conductivity, and lack of active surface sites [58]. Various strategies have been adopted to address these issues by modifying the 2D materials through surface defects engineering, heteroatom doping, surface functionalization, and increasing interlayer spacing by intercalation and hybrid structures. MXene-based materials are used in high-performance supercapacitors due to their high conductivity, intercalation of polar organic molecules and metal ions, and facile adsorption of ions due to surface functional groups (–O, –OH, –F) [59]. Xia et al. demonstrated the capacitive performance of vertically aligned Ti_3_C_2_T_x_ MXene films by mechanical shearing. The vertical alignment of MXene sheets led to facile ion transport, and the capacitance was retained up to 200 Fg^−1^ at 2000 mVs^−1^ (independent of the thickness of the film) [60]. Zhao et al. prepared sandwich and micro-sized supercapacitor electrodes using synchronous reduction and self-assembly of MXene on Zn foil. Due to the high electrical conductivity porous architecture, the devices showed excellent electrochemical performance even in bending states, demonstrating the application of MXenes for flexible and wearable energy sources [61]. 2D Transition Metal Oxides (TMOs) are another exciting class of electrode materials with high specific capacitance and energy density due to reversible redox reactions [62]. Some of the widely used metal oxides for supercapacitor electrodes include MnO_2_, NiCo_2_O_4_, RuO_2_, Fe_3_O_4_, and NiO. Though metal oxides have a high specific capacitance of 200–2000 Fg^−1^, they suffer from poor electrical conductivity, cracking of electrodes during cycling, and limited access due to varied coordination. Ma et al. investigated the performance of MnO_x_ nanosheets on MnO_x_@Carbon nanowire synthesized by liquid phase method with thermal treatment [63]. MnO_x_ nanosheets offer abundant surface-active sites for electrochemical activity. The all-solid-state supercapacitor fabricated using nanosheets of MnO_x_ show a maximum power density of 2500 W kg^−1^ and energy density of 23 Wh kg^−1^ with 94% capacitance retention up to 2000 cycles [63]. The effect of defect engineering on 2D materials was explored by Chen et al. using amorphous Vanadium Oxide nanosheets for asymmetric supercapacitors. The defects increased the oxygen vacancy content to 28.5%, facilitating ion migration and leading to the ultra-high specific capacitance of 554 mF cm^−1^ at 1 mA cm^−2^ [64]. Santos et al. reported a cost-effective, single-step synthesis of transition metal oxide (Co and Cu) grafted carbon nitride nanosheets. The asymmetric supercapacitor fabricated using Co and Cu oxides hybrid exhibited a specific capacitance of 124.5 F g^−1^ and 84.28 F g^−1^ with cycling stability of 96% retention after 2000 cycles [65]. Transition metal dichalcogenides (TMDs) finds application in supercapacitor due to their large surface area and rapid redox reactions at the surface but possess low capacitance retention. Some of the widely used TMDs include MoS_2_, WS_2_, TiS_2_, NbS_2_, and VS_2_. In Fig. 5a, Adhikari et al. reported WS_2_-PANI composite, which exhibited excellent high-frequency supercapacitor. WS_2_-PANI composite exhibited the capacitance of 72.27 Fg^−1^ at 1 Ag^−1^ and a high-frequency response of 6 kHz. The device exhibits cyclic stability of 85% and coulombic efficiency of ~ 100% in Fig. 5b [66]. Some other novel 2D materials such as phosphorene, silicone, bismuth and, stannene, have been explored for supercapacitor application due to their properties analogous to graphene. [67] Kim et al. performed the first polymerization of 2D Phosphorene using conducting polypyrrole. The specific capacitance of hybrid was reported to be 411 Fg^−1^ which is four times as compared to polypyrrole as electrode material [68]. Phosphorene is a good is electrode materials that are highly hygroscopic and sensitive to ambient atmosphere. In addition, various transition metal oxides and carbon-based are used as electrode materials but they lack due to poor conductivity and low density, respectively. It been observed that the 2D layered materials with sheets arranged in specific manner in crystals are best since it offers high surface exposure for reaction as compared to other conventional electrode material [69]. Graphene, porous carbon sheets, and carbon nanotube are well reported for their supercapacitive application and commercialization. Graphene is 2D layered material with excellent mechanical strength, high surface area, and, high conductivity due to sp^2^ hybridized carbons in 2D sheets structure. Despite this, graphene nanosheets undergo restacking decreasing electrolyte charge distribution at the electrode surface. To overcome this issue, chemical functionalization of graphene/graphene oxide with heteroatoms, intercalation, etc. has been done for better supercapacitive performance [69, 70]. Robert A. W. Dryfe and coworkers prepared N-doped graphene oxide with hexamethylenetetramine by the hydrothermal process followed by thermal expansion resulting in a high specific capacitance value of 270 F/g at 1 A/g [71]. Park et al. used 2D controlled oxidized black phosphorous covalently bonded to graphene and achieved a high capacitance value of 478 F/g [72]. To fulfill the modern need for flexible and portable energy devices, flexible supercapacitors are developed. Yan Li and their group made a flexible solid supercapacitor by coating gel electrolyte and N-dopped CNT/GO composite to both sides of the paper. A high volumetric capacitance value of 0.45 F/cm^3^ was reported and an energy density of 40.0 μWh/cm^3^ [73]. Zheng and coworkers prepared a 2D hierarchical porous carbon nanosheet using polyaniline with acetaldehyde followed by carbonization and activation by KOH and achieved a volumetric capacitance of 3.8 F/cm^3^ with a high energy density of 8.4 mWh/cm^3^ [74]. Chong et al. synthesized MoS_2_/graphene composite by electrochemical etching of bulk MoS_2_ and graphite electrode to prevent restacking of MoS_2_ for supercapacitor with a high specific capacitance of 227 F/g and capacitance retention up to 89% over 2500 cycles [75]. Gupta and co-workers developed a catalytic approach to convert waste polystyrene into highly conducting graphitic carbon sheets with high surface area for electric double-layer supercapacitors with a high specific capacitance of 158 Fg^−1^, linear response with scan rate, and 90% capacitance retention after 10,000 cycles. [76] Gordon G. Wallace and the group synthesized MoS_2_/PEDOT: PSS (MP) hydrogel by the one-pot hydrothermal process by controlling the growth of MoS_2_ on the PEDOT surface. The supercapacitor device with solid-state electrolyte PVA–H_3_PO_4_ resulted in a high capacitance of 360 mF cm^−2^ at 0.5 mA cm^−2^ with capacitance retention of 89% [77]. Li et al. prepared a composite of oxygen vacancy-rich NiCoO_4_ with nitrogen-deficient graphitic carbon nitride by one-pot heat treatment in presence of air. The high specific capacitance of 1998 F g^−1^ at a current density of 2 A g^−1^ was achieved with 95.22% capacitive retention after 5000 cycles [78]. Nayak and group synthesized a composite of SnS_2_ with oxygen functionalized graphitic carbon nitride and CNT by a hydrothermal process resulting in a high specific capacitance of 433 F/g and 101% capacitance retention over 7000 cycles [79]. Sundramoorthy and group synthesized free-standing flexible electrode with a composite of hexagonal boron nitride and graphene paper by vacuum filtration of a mixture of h-BN and graphene nanosheets dispersions achieving excellent specific capacitance of 321.95 F/g at a current density of 0.5 A/g with 96.3% capacitance retention after 6000 cycles [80]. Dai and his group prepared alternative stacked 2D/2D MXene/ZnMnNi layered double hydroxides through electrostatic self-assembly of negatively charged MXene surface and positively charged ZnMnNi LDH surface by a simple string of their water dispersions, which showed capacitance value of 2065 F/g at a scan rate of 5 mV/s and with capacitance retention of 99.8% after 100,000 cycles at a current density of 1 A/g [81]. NPs were also used as an interlayer spacer for pseudocapacitive performance. For example, Warsi and coworkers intercalated CoFe_2_O_4_ NPs to prepare CoFe_2_O_4_/ MXene composite providing much higher capacitance (1268 F/g) and 97% capacitance retention up to 5000 cycles [82]. Rout et al. synthesized a composite of 1 T phase of VS_2_ nano-flower with 2D MXene by in-situ hydrothermal method to achieve a high specific capacitance value of 115.7 F/g at a current density of 0.8 A/g with 85% capacitance retention over 5000 cycles, as well as the highest energy density achieved was 41.13 W h/kg [83].

### 2D materials for Gas/VOC and humidity sensors

**Fig. 6:**
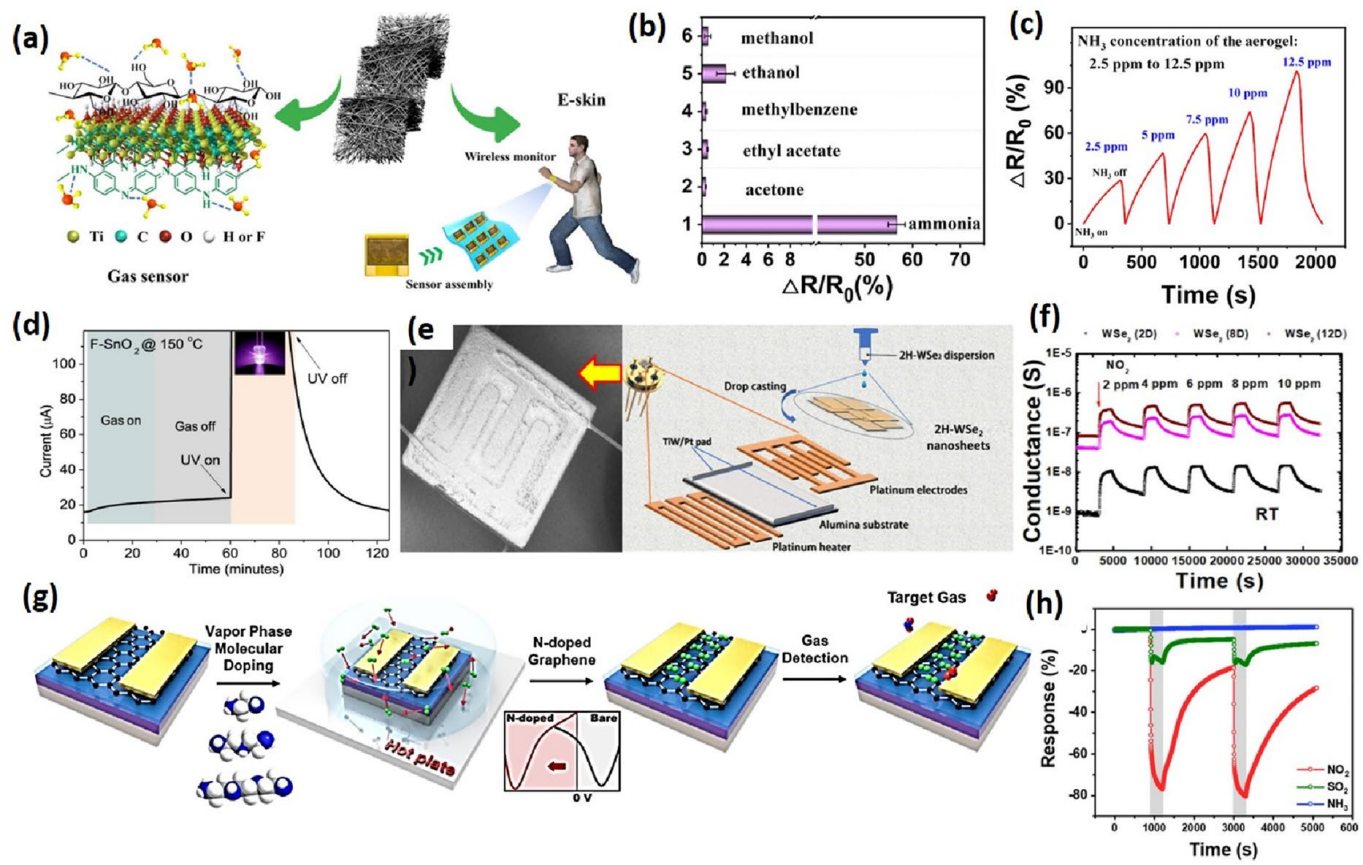
**a** Components of the MXene/PANI/BC aerogel and the schematic of the MXene/PANI/BC aerogel for the applications of e-skins and gas sensors. **b** Selectivity. **c** Response of the Ti_3_C_2_T_x_ MXene/PANI/BC composite aerogel-based gas sensor to NH_3_ gas for varied concentration ranges. [98] **d** Sensor current response profile with the UV-on of F-SnO_2_ sensor [101] **e** fabrication process and design of the 2H-WSe_2_ nanosheet sensor. **f** Dynamic response of 2H-WSe_2_ (2D), 2H-WSe_2_ (8D), and 2H-WSe_2_ (12D) sensors at RT. Figure adapted from ref [109] **g** Schematic illustration of the fabrication process of a FET gas sensor based on n-doped graphene **h** Gas sensing characteristics of DETA-doped graphene [102]

Development in industrialization has led to a rapid rise in air pollution due to toxic gases, smoke, and emission, which pose a serious threat to human health. Various indoor air pollutants in volatile organic compounds (VOCs) come from paints, cosmetics, furnishing, glues and adhesives, cleaning and disinfecting products, etc. Exposure to toxic gases/VOCs higher than the maximum permissible limit or prolonged exposure causes severe health problems. Thus, gas and VOC sensors that can detect and monitor toxic gases in the environment have become extremely important for environmental monitoring. They are also a potential candidate for healthcare applications for detecting various biomarkers for disease diagnosis. The concentration of VOCs in human breath act as biomarkers for different disease such as toluene for lung cancer, acetone for diabetes, ammonia for renal failure, nitric oxide for asthma, etc. However, detecting the gas/VOCs at ppb level in a varying humidity atmosphere is extremely challenging. There is a dire need to develop sensing materials that are highly specific and sensitive toward selective molecules even under humid conditions [88]. For real-time monitoring and industrial application, the gas sensors need to be highly responsive, selective, have low fabrication cost, and have low power consumption. 2D materials have proven highly efficient due to their fascinating chemical and physical characteristics. Prospects for 2D materials include stability of the material in an ambient atmosphere with room-temperature operation for practical application. A diverse range of 2D materials has been explored for gas sensing, including carbon nanomaterials, layered metal oxides, transition metal dichalcogenides, conducting polymers, MXenes, Borophene, carbon-based 2D materials, etc. [89]. These materials have advantages and disadvantages, such as high operating temperature, low conductivity, and low surface area. 2D materials, due to their unique physical and chemical property, mechanical flexibility, and high electron mobility, are widely used for gas sensing applications [88, 89]. The gas-sensing performance depends on various parameters such as sensitivity, selectivity, stability response/recovery time, detection limit, reproducibility, and reversibility. 2D layered materials offer high specific surface area and tremendous reactive adsorption sites leading to high sensitivity due to facile analyte-material interaction. Moreover, higher conductivity leads to low noise and results in relatively lower Limits of Detection. Owing to tunable surface functionalization in 2D material, they provide high selectivity by tuning the surface in a way to provides strong affinity towards the target gas. As the surface of 2D materials is highly sensitive, even a small change in the physical parameter of sensing material will lead to a detectable signal and hence provide good reversibility [90]. A large surface-to-volume ratio and tunable surface endow 2D materials with excellent properties for sensing application. Tailoring the morphology and surface structure by novel metal ion doping or heterostructure formation can significantly improve the sensing performance [91]. When 2D materials with different electrical properties come in contact, they form heterojunction at the boundary by carrier diffusion and Fermi-level alignment. The depletion layer formed at the junction has high resistance, changing when interacting with the gaseous analyte. The heterostructure of 2D materials includes metal–semiconductor which gives rise to the Schottky barrier or semiconductor–semiconductor junction (p-p, p-n, and n-n heterojunction) [91]. The gas-sensing performance can be improved by making 2D heterostructures with 0D, 1D, 2D, and 2D nanomaterials. Moreover, several heterostructures have been reported in the literature for enhanced sensing performance, but which electronic and chemical effect leads to which type of sense has not been fully explored and explained yet. Therefore there is a need to develop an insight into the typical chemical and electronic changes caused by the type of heterostructures for predicting the sensor behavior towards a specific analyte [91]. Among all layered materials, 2D Transition Metal Oxides (TMOs) are most extensively being utilized for sensing applications due to reduced bonding coordination and high surface polarization with fascinating chemical and physical properties than their bulk counterparts. Metal Oxides have pre-adsorbed oxygen on the surface which leads to the formation of an electron depletion layer on the surface. When an analyte interacts with the surface, depending on the nature of the interacting analyte it will either increase or decrease the thickness of the depletion region and hence change the resistance of the material. Metal oxides nanosheets have a large specific surface area and thickness equal to Debye length. They have strong in-plane bonds and weak van der Waals interaction between layers [92]. The adsorption of a gas on metal oxide act as either p-type or n-type dopants depending on oxidizing or reducing nature of the gas, leading to a change in carrier concentration and hence changing the conductivity of the material. Metal Oxides work in an aerobic environment at high temperatures. Some commonly used 2D metal oxides for gas sensing applications include SnO_2_, WO_3_, MoO_3_, TiO_2,_ and their composites. Despite various advantages of metal oxides for gas sensing, they suffer from high operating temperature, which can be addressed by using 2D materials with heterostructures. Wang et al. fabricated a sensor using Graphene oxide/SnO_2_ nanosheets with a response as high as 2000 towards HCHO. The excellent performance of the sensor was due to the synergistic effect of the sensitizer effect of GO, ultrathin nanostructure, and in-plane mesopores which are conducive to gas diffusion [93]. Qin et al. synthesized Co-based bimetallic metal oxide nanosheets for CO sensing. The sheets possess an ultra-high surface area of 220.7 m^2^ g^−1^, exposing abundant active sites. The sensor exhibited ppb level detection with room-temperature operation [94]. Jang et al. developed subatomic level thick heterogeneous oxide SnO_2_/CoO_x_ nanosheets for gas sensing. The heterogeneous oxide was synthesized using an exfoliation approach and a Galvanic replacement reaction. The GRR process leads to the formation of plentiful SnO_2_/CoO_x_ heterojunction with high porosity and sub-10 nm thickness which are highly desirable for gas sensors. The sensor also showed exceptional selectivity towards formaldehyde gas at 5 ppm with recovery in < 10 s. The synthesis procedure adopted also opens ways for scalable production of heterogeneous oxides for sensing applications [95]. Various MXene-based hybrid materials have been reported in the literature for sensor fabrication for different gas/VOCs. Sun et al. reported a susceptible and selective NO_x_ sensor using Co_3_O_4_@PEI/Ti_3_C_2_T_x_ nanocomposite with a low limit of detection of 30 ppb at RT. The heterojunction between p-type Co_3_O_4_ and conducting MXene provides facile electron transport for NO_2_ sensing [96]. In Fig. 6a–c, Zhei et al. demonstrated a flexible pressure and gas sensor using MXene/PANI/ bacterial cellulose aerogel with high selectivity toward NH_3_. The 3D structure improved the synergetic effect and enhanced the electron transfer due to improved gas adsorption and diffusion. The H-bonding between MXene, PANI, and BC provided stability to channels. The LOD value was determined to be 56.49 ppb [97]. Kim et al. reported a self-assembly method to intercalate metal ions in MXene forming transparent films that were utilized as gas sensors to detect ammonia. The intercalation improved the signal-to-noise ratio to tenfold compared to pristine MXene. The sensing performance of Na, K, Mg, and Ca intercalated MXene was compared, out of which Na-MXene showed the highest response with LOD of 10 ppm due to low film thickness and high electrical resistance [98]. He et al. developed a gas sensor based on MXene/SnO_2_ heterojunction for ammonia sensing at RT. The difference in Fermi level of SnO_2_ and MXene enhanced the sensitivity by facile charge transfer at the interface. The sensor showed excellent sensitivity from 0.5 ppm to 100 ppm ammonia with a 20 times higher response to 100 ppm ammonia [99]. Liu et al. fabricated a gas sensor using α-Fe_2_O_3_ nanocubes/Ti_3_C_2_Tx composite with excellent selectivity towards acetone. The α-Fe_2_O_3_ nanocubes attached to the MXene sheets by electrostatic interaction provided a higher active surface area for gas adsorption. The sensor showed linear response from 5 to 200 ppm acetone concentration with rapid response and recovery time of 5 s [100]. In Fig. 6d, Bahuguna et al. fabricated a thin-film F-SnO_2_-based sensor for VOC detection [101]. In Fig. 6g, h Kwon et al. reported an N-Channel graphene sensor for NO_2_ detection with a detection limit as low as 0.83 ppm [102]. Borophene, with its high electronic conductivity and large surface area, finds application in gas sensing [103]. Hou et al. reported the first NO_2_ gas sensor based on borophene with high sensitivity, fast response, high selectivity, and good stability. The sensor also showed good stability for up to 1000 bending cycles, making it suitable for wearable electronics. Although borophene-based sensor shows a strong response to NO_2_ the sensitivity is still lower than carbon nanomaterials and polymer composites [104]. Another research, conducted by the same group, demonstrated the graphene-borophene heterostructure for humidity sensing. The sensor showed ultrahigh sensitivity, almost 700 times higher than graphene. The sensitivity towards humidity was as high as 4200% at 85% RH, the highest among all 2D materials [105]. Shen et al. theoretically investigated the adsorption behavior of organic molecules CH_4_, C_2_H_4_, C_2_H_2_, CH_3_OH, and HCHO on Borophene and borophene/MoS_2_ heterostructure. C_2_H_2_ and HCHO show strong interaction with borophene and the heterostructure shows high sensitivity towards these molecules [106]. Various computational studies reveal that Borophene is an emerging class of 2D nanomaterials and has much potential for gas sensing applications, but experimental research is still very limited. For gas/VOC sensing, the performance of Transition Metal Dichalcogenides (TMDs) surface structure modifications, defect engineering, exposed edge sites, and doping have been explored. Pham et al. fabricated a MoS_2_-based optoelectronic NO_2_ sensor using graphene. The sensing performance was enhanced by inducing photocurrent. The sensor showed high sensitivity and low limit of detection upto 0.1 ppb. TMDs-based gas sensors have sluggish recovery and have cross-sensitivity towards multiple analytes [107]. Ko et al. worked on enhancing of recovery performance of WSe_2_-based gas sensors. The sensor showed excellent sensing performance towards NO_2_ with a response value of 4140%. The recovery was improved by using external thermal energy and by reacting NH_3_ with adsorbed NO_2_ on the surface which decreased the recovery time from 85 min to 43 s [108].

### Electrochemical sensors

Modern chemical sensors use various features to determine biological, physical, and chemical aspects in day-to-day life [110]. Some examples are health sensors, environmental monitoring, sensors for instrumentation, and sensors related to machineries like airplanes, automobiles, and mobile phones [111]. Electrochemical sensors measure the electrochemical reactions and determine them with the help of electrodes due to the analyte and the sensing surface interactions, converting the information into quantitative and qualitative electric signals based on potentiometry, conductometry, and amperometry measurements [112]. The signal is measured by detecting changes in the faradaic current or interfacial resistances. The mechanism involves the usage of redox mediators to determine the capturing of a specifically targeted molecule onto the working electrode. The redox mediator accessible surface area decreases as the target analyte is captured, resulting in a drop in electrochemical current or a rise in interface resistance [113]. The development of novel 2D nanomaterials for working electrodes is crucial in advancing electrochemical sensors. Two-dimensional layered materials exhibit a high value of surface-area-to-volume ratios for analyte interaction and a very high environmental sensitivity, which are important for chemical sensing applications [114]. Furthermore, 2D-nanomaterials possess excellent resilience and flexibility, making them capable of developing a new class of high-performance flexible electrochemical sensors [115]. By facilitating the electron transfer process and signal creation, 2D layered materials in electrochemical sensor devices seek to improve sensitivity and performance. Electrochemical sensors have the potential to determine analytes at much less concentration than state-of-the-art sensing technologies [116]. For example, Rahman et al. fabricated a sensing platform based on graphene oxide and silver nanowire nanocomposite for improved sensing performance of Hg^2+^ [117]. Choi et al. fabricated a graphene oxide doped diaminoterthiophene-based sensor to simultaneously determine copper, cadmium, mercury, and lead at a very low concentration [118]. Different chemical sensors have been developed based on 2D materials for various analytes that must be monitored, such as toxic metal ions, pesticides, herbicides, soil, and other important pharmaceutical compounds, bio-molecules like glucose, dopamine, uric acid, and cholesterol [119, 120]. For example, Murugan et al. developed an electrochemical sensor to simultaneously detect uric acid, dopamine, and ascorbic acid. The detection limit was 0.06 μM, 4.6 μM, and 0.075 μM for dopamine, ascorbic acid, and uric acid, respectively [121]. Ribeiro et al. fabricated an enzyme-free electrochemical sensor using an rGO and cobalt oxide hybrid material recycled from Li-ion batteries to determine ascorbic acid [122]. Also, conventional materials are used for the nonenzymatic-based determination of glucose; however, it is still challenging to detect it at a neutral pH. Many of the previous reports for the detection of glucose use highly alkaline solutions, which are difficult to implement for continuous monitoring and wearable systems. By incorporating 2D materials and improving the sensitivity, sensors for glucose determination in the neutral electrolyte have been developed. For example, Chen et al. reported a glucose sensor based on a nonenzymatic method at a neutral pH using graphene nanosheets modified with Pt–Pd nanocubes covered electrode [123]. In Fig. 6e, f, Moumen et al. fabricated a NO_2_ sensor with a 2H variant of tungsten diselenide with a 361% response to 6 ppm NO_2_.[55] Foroughi et al. developed graphene-modified CuO nanoparticles for glucose sensing at biological pH [124]. Hybrids of 2D layered materials and their heterostructures utilize transition metal redox centers for interaction with the analytes and fabrication of multiplexed sensing systems. Reactive nitrogen species (NS) and Oxygen species (OS), such as NO or H_2_O_2,_ are important molecules involved in cellular metabolism and several signaling processes inside the body [125]. A nitrosative and oxidative stress can occur if the concentrations of NS/RO are left unbalanced by the antioxidant system in the body, and long-term stress can cause various diseases such as Alzheimer’s, Parkinson’s, and cancer [126, 127]. Therefore, proper continuous monitoring of their concentrations becomes important for identifying disease and good health. Electrochemical detection of heavy metal ions (HMIs) at ppm and ppb levels depends on the electrochemical reactions occurring due to metal ions at the interface of the electrode and the electrolyte solution resulting in an electrical signal. As the HMIs have specified redox potentials, selective determination of certain HMIs can be obtained without using a molecular identification probe utilizing bare electrodes. The anodic stripping voltammetry (ASV) approach, in particular, is being investigated for heavy metal identification. In this, the heavy metals are deposited on the electrode surface followed by stripping or dissolving of the analytes, which are deposited on the electrode surface, which are the two phases in ASV analysis. For example, Wei et al. fabricated a sensor based on SnO_2_/graphene oxide nanocomposite for the detection of Hg(II), Cd(II), Cu(II), and Pb(II) in drinking water [128]. Neog et al. developed a WS_2_ nanosheets-based sensor to detect Zn(II) [129]. Hui et al. developed a titanium carbide (Ti_3_C_2_Tx) and multiwalled-carbon-nanotubes (MWNTs) nanocomposites based sensor to detect Zn(II) and Cu(II) ions [30]. Pifferi et al. fabricated a sensor based on rGO sheets, functionalized surface with 1-pyrene carboxylic acid, and loaded in situ by gold nanoparticles to determine As(III) ion [130]. A traditional electrochemical sensor has a single response signal, and this has limited the reproducibility due to a variety of parameters such as different properties of the base electrode (i.e., area, morphology), the density of probe loading, and complex detection environments. Due to the virtues of high interference and better reproducibility, the ratiometric technique, a detection mode commonly used in fluorescence research, has been utilized in electrochemical sensors to overcome these challenges [131]. In contrast to traditional single-signal electrochemical sensors, ratiometric electrochemical sensors (RESs) possess two electrochemical signals and utilize the ratio between the two signals as the output signal rather than the single signal absolute value. Therefore, RESs require precise dual electrochemical signal selection, which is a significant design aspect [132]. Many RESs based on 2D materials have been developed to determine various analytes. For example, Xu et al. constructed a ratiometric-antifouling electrochemical biosensor based on multifunctional peptides and MXene with gold nanoparticles and methylene blue for detecting prostate-specific antigen and thrombin [133]. Qu et al. developed a ratiometric electrochemical platform for detecting ascorbic acid with excellent selectivity, high sensitivity, and good reproducibility by tailoring oxygen-containing groups on graphene [134]. Wang et al. developed a ratiometric electrochemical immuno-sensor for sensitive determination of Human epidermal growth factor receptor-2 using reduced graphene oxide and polydopamine grafted ferrocene/Au@Ag nanoshuttles performing as an electrode material and a hollow Ni@PtNi yolk-shell nanocages-thionine as the signal tags, which measured distinct electrochemical signals for Fc and Thi, respectively [135]. Zhong et al. used MXene on Ag nanoclusters and amino-functionalized multi-walled carbon nanotubes composite to create a new ratiometric electrochemical sensor for the detection of carbendazim [136]. Urgunde et al. performed simultaneous detection of dopamine and uric acid using NiCo_2_O_4_ on a printed biosensor chip with low LOD and high sensitivity [33].

**Fig. 7:**
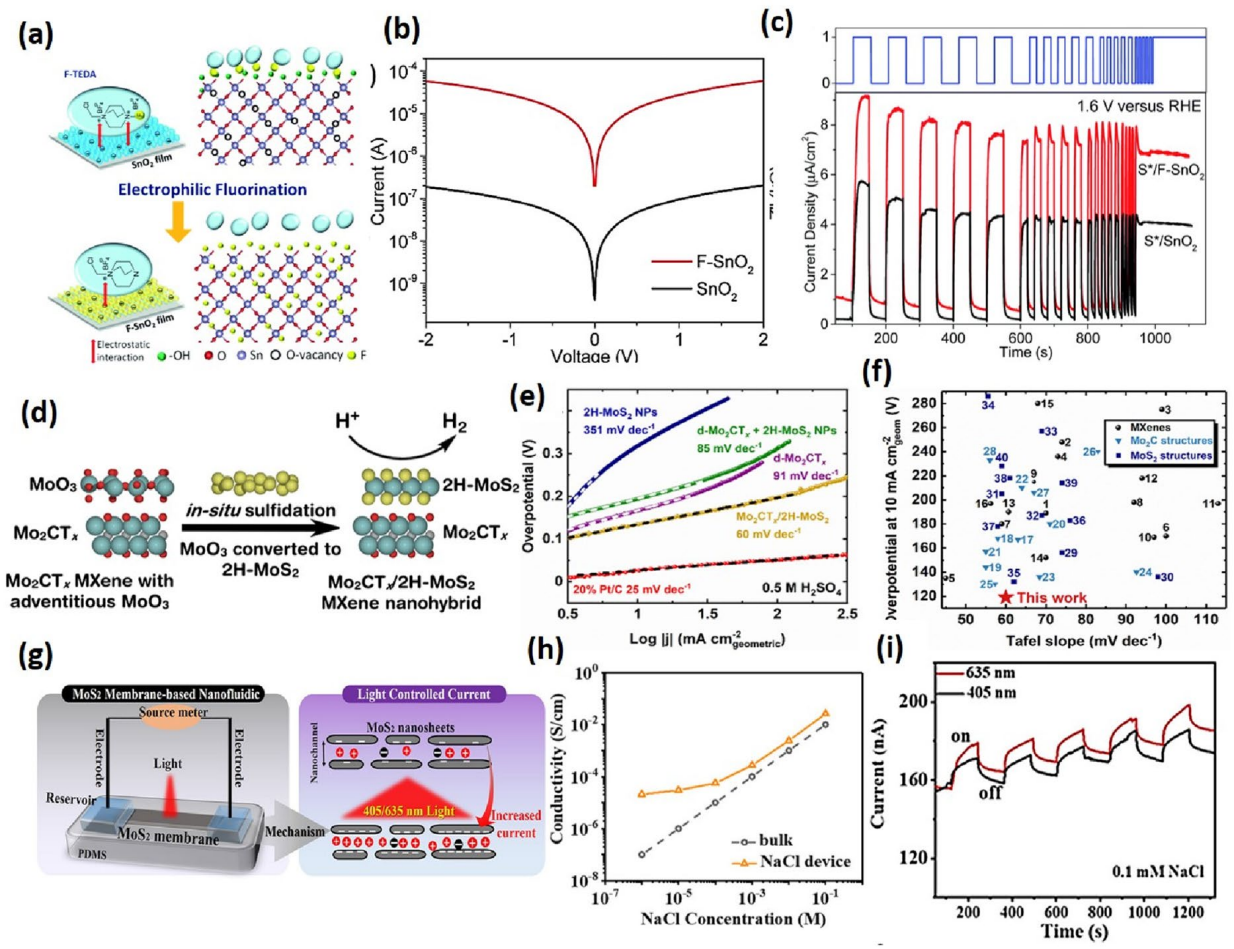
**a** Schematic demonstrating the fluorination process of the SnO_2_ film using the F-TEDA molecule as the fluorinating precursor. **b** I–V characteristics of SnO_2_ and F–SnO_2_ films, **c** J–t measurements of S*/SnO_2_ and S*/F–SnO_2_. Figure adapted from ref [142] **d** Schematic representation of in situ sulfidations produce Mo2CTx/2H-MoS2 nanohybrid, **e** Tafel plot, **f** HER benchmarking of Mo2CTx/2H-MoS2 against other material [162] **g** Schematic illustration of ion transport through the nanochannel in the MoS2 membrane **h** Ionic conductivity of the MoS2 membrane in NaCl solution **i** Reversible switch of the ion transport under 405 and 630 nm light illumination in the NaCl solutions [163]

Different types of 2D materials are widely used for varying electrochemical applications. Among various layered 2D materials, Graphene is highly explored for many electrochemical applications due to its unique properties. It is a one atom thick layer comprising six sp^2^ carbon atoms connected in a honeycomb fashion. Usually, it is used in the pristine form as a support system due to its high surface area and conductivity. It is also used in Graphene Oxide (GO) and reduced form as reduced Graphene Oxide (rGO). GO has the advantage of forming suitable dispersions in polar solvents due to polar oxygen functional groups. But this also leads to decreased electrical conductivity. Direct application of graphene suffers from issues like agglomeration and low sensitivity. Therefore, nanomaterials-based graphene systems are used to overcome these problems. Various graphene and its derivatives-based electrochemical sensors have been reported throughout the literature. For example, Santos et al. developed a laser-induced graphene-based sensor for the selective and sensitive detection of dopamine [137]. Recently Xian et al. reported a Zr-based MOF and graphene composite to determine Ciprofloxacin (CIP) in water by depositing Cu^2+^ ions on the electrode. In the presence of Cip, a complex is formed with Cu^2+^, which results in the decrease of the oxidation current of Cu ions. A limit of detection of 6.67 nM was reported with an excellent linear range of 0.02–1 μM [138]. Graphene oxide nanocomposites can also be utilized in the selective detection of HMIs because of the affinity of metal ions to form bonds with -COOH groups present on the Graphene oxide surface. Rahman et al. used a functionalized graphene oxide and Ag nanowire to detect Hg^2+^. The sensor demonstrated excellent selectivity towards Hg^2+^ ions even in the presence of Cd^2+^, Pb^2+^, Na^+^, Cu^2+^, and Ag^+^. Hg^2+^ has a higher reduction potential, resulting in easy bond formation with –COOH groups. Therefore the electrode was highly selective towards Hg^2+^ ions with a detection limit of ~ 0.1 nM with a linear response of 1–70 nM and high sensitivity of 0.29 μA/nM [63]. Scandurra et al. also fabricated a low-cost disposable sensor of Graphene Paper-Nafion-Bi Nanostructures for Ultra-Trace detection of Pb^2+^ and Cd^2+^. The sulfonic groups in Nafion get reduced partially to -SH and organic-sulfides during electrodeposition of Bi. These negatively charged sulfonic groups with reduced thiol groups help bind Pb^2+^ and Cd^2+^ ions, improving the limit of detection. A LOD of 0.1 ppb with a 5–100 ppb linear range was reported [139]. Pizzaro et al. fabricated a green and inexpensive electrochemical sensor using graphene quantum dots and Nafion to determine Cd^2+^ and Pb^2+^. The selective permeability of positive ions of Nafion, with the high number of active sites due to graphene QDs, also results in an excellent electrochemical response with LOD of 8.49 μg L^−1^ for Pb^2+^ and 11.30 μg L^−1^ for Cd^2+^ with a linear range of 20–200 μg L^−1^ [140]. Deshmukh et al. reported a Red Mud-Reduced Graphene Oxide-based electrochemical sensor for Arsenic detection. An excellent limit of detection of 0.07 ppb with a linear range of 0.1–2.3 ppb was found. These results can be attributed to the superb absorption proficiency of hematite-rich Red mud [141]. In Fig. 7a–c Bahuguna et al. performed single-step electrophilic fluorination of SnO_2_ for photoelectrochemical application [142]. Borophene is a non-planar buckled molecule with hexagonal vacancies. Limited literature is available due to its toxic starting material, diborane. It is considered superior to graphene in flexibility, strength, and conductivity. Borophene is postulated as a promising material for gas sensing applications. A lot of computational studies have been done in this area. Recently Shukla et al. presented a computational study for sensing various gases, including CO, NO, NO_2_, NH_3_, and CO_2_. They reported good binding energies for all gases except CO_2_ [143]. More computational literature can be found for other molecules like formaldehyde [144] and hydrogen cyanide [145]. Some recent publications have also reported a few non-enzyme borophene-based biosensors. Güngör et al. have reported a Copper phthalocyanine-borophene nanocomposite for urea sensing with a detection limit of 0.05 μM in a linear range of (250–1000) μM [146]. Taşaltın reported a sensor for glucose based on PAN: borophene composite with a detection limit of 0.099 mM [147]. Borophene has a lot of potential in gas sensing applications looking at current computational studies. But due to its susceptibility to oxidation at ambient conditions and toxic starting materials, research is still limited.

Among 2D materials, metal, non-metal and metal-free nitrides are also used in electrochemical sensing due to their good chemical stability, availability, electronic structure, and adjustable bandgap. Among non-metal nitrides, Graphitic carbon nitride (GCN) is quite studied, and many researchers have applied it for heavy metal ion detection [148]. Wang et al. reported porous form S-doped C_3_N_4_ tubes/ graphene nanosheets to detect Cd^2+^, Hg^2+^, and Pb^2+^ electrochemically. Good LOD was observed due to synergy between hierarchical porous tubes and sulfur doping. The LOD was 1.17, 0.61, and 0.38 nM for simultaneous detection of Cd^2+^, Hg^2+^ and Pb^2+^. Chen et al. used AuNPs/mpg-C_3_N_4_ nanocomposite to detect methylmercury. Here GCN provides a large surface area for adhesion of gold nanoparticles providing good LOD values [149]. Kokulnathan et al. used Titanium Carbide/Boron Nitride Nanocomposite for sulfadiazine sensing. Low charge transfer resistance (20 ohms), good peak potential separation (0.080 V), and good surface area (0.106 cm^2^) were observed. LOD of 3 nM was reported [150]. Chen et al. used defect engineering to detect H_2_O_2_ using self-assembled PGP (polycrystalline GaN porous layers). Self-assembly at low temperature resulted in N-vacancies which improves electrical conductivity. LOD of 50 nM was reported with a fast response speed of 3 s [151]. Liu et al. grew n-type GaN micropillar on p-type GaN substrate electrodes to detect Ag^+^ and Cu^2+^ ions simultaneously. LOD of 3.3 ppb for both ions was reported [152]. MXene is another 2D material with unique properties in sensing applications. In Fig. 5d, f, Park et al. used Ti_3_C_2_T_x_ with MWCNTs for Cu and Zn detection. The improved performance was attributed to the increased surface incorporating the MWNTs as anti-pile layers, which removed the aggregation problem between Ti_3_C_2_T_x_ layers and led to a more exposed surface area. In situ Sb deposition was also performed to improve the detection limit further. Low detection limits of 1.5 ppb and 0.1 ppb were reported for Zn and Cu, respectively [30]. Xu et al. used functionalized peptides with Mxene decorated with methylene blue and Au nanoparticles to develop ratiometric antifouling electrochemical biosensors. Accurate prostate-specific antigen detection was reported with a limit of detection of 0.83 pg/ml and a detection range of 5 pg/ml to 10 ng/ml [79]. Yi et al. used Ti_3_C_2_T_x_ nanoribbons for HMIs detection using Cd^2+^ as a representative element. A self-reduction strategy was used in place of electrodeposition in the ASV sensing. A low LOD of 0.94 nM with a wide linear range of 0.005–3.0 µM was reported [153]. Xu et al. reported HRP/Ti_3_C_2_/Nafion film detecting H_2_O_2_ in patients’ serum samples with acute myocardial infarction. A catalytic effect of HRP on H_2_O_2_ was observed. LOD of 1 μM and a linear range of 5–8000 μM [154]. Gul et al. developed a 2D Nb_2_C MXene-based sensor for hydrazine electrochemical sensing [155]. Rasheed et al. reported a sensor based on Pd@Ti_3_C_2_T_x_ nanocomposite to detect L-cysteine. The MXene acted as a conductive matrix and a reducing agent at the surface of the electrode, and PDNPs improved the stability of Ti_3_C_2_T_x_. The detection limit of 0.14 μM with a linear range of 0.5–10 μM [156].

Transition metal dichalcogenides represent another class of layered materials used in electrochemical sensing. MoS_2_ has been widely studied in this area due to its structural properties. Hwang et al. reported electrochemical detection of Pb^2+^ using MoS_2_ nanofilms. Vertical alignment of MoS_2_ films performed better as compared to horizontally aligned MoS_2_. This sensor showed excellent sensitivity towards Pb^2+^ concentration with an improved LOD of 0.3 ppb with a linear range of 0–20 ppb [157]. Lee et al. reported a sensor based on MoS_2_ and graphene composite to detect morin electrochemically. Graphene provides a support system to increase active surface area to increase electrochemical performance [158]. Yuan et al. reported a NiS_2_ microblock, MoS_2_ nanosheets, and rGO for electrochemical detection of Bisphenol-A. The synergy effects of NiS_2_/MoS_2_/rGO provided increased conductivity and sensitivity. A lower detection limit of 2.1 nM with a linear range of 0.02–200 μM was reported [159]. Zhou et al. used defect and phase-engineered Mn/ MoS_2_ nanosheets for heavy metal ion detection. Mn- mediation resulted in a new defect-rich 1 T-MoS_2_ form 2H-MoS_2_, with better electrical conductivity [160]. References using VS_2_ and WS_2_ have also been reported in the literature. Yuan et al. developed 2D VS_2_/VC/N-Doped carbon sheets loaded with ultrafine-Pd nanoparticles for H_2_O_2_ sensing. These particles were assembled on carbon-fiber microelectrodes in a 3D array. This setup improved the electron transfer ability, stability, electrocatalytic activity, and biocompatibility with a LOD of 50 nM [161].

### Electrocatalysis

Currently, research for the development of electrocatalysis using 2D layered materials has been increasing. Some of the emerging 2D layered materials for electrocatalysis include metal oxides (MO_x_), nitrides of carbon and boron (CN/BN), 2D metal–organic frameworks (MOFs), transition-metal chalcogenides/dichalcogenides (TMCs/TMDs), 2D covalent organic frameworks (COFs), black phosphorus, MXenes, and layered double hydroxides (LDHs). Compared to their bulk equivalents, the 2D materials show some exceptional properties. These nanosheets possess extraordinary physiochemical properties such as high specific surface area, tunable active sites, etc., enabling them to serve as promising electrocatalysts for different electrochemical processes.

*Electrochemical Water Splitting (HER and OER)* In Fig. 7d, f, Kang Lim et al. demonstrated that Mo_2_CT_x_/2H-MoS_2_ nanohybrids are prepared by an in-situ process via a two-step mechanism sulfidation occurs and the extrinsic oxide layers on Mo_2_CT_x_ are converted to 2H-MoS_2_. This strongly-coupled nanohybrid electrocatalyst shows enhanced HER performance at − 10 mA cm^−2^ current density in 0.5 M H_2_SO_4,_ requiring only 119 mV overpotential [162]. Zepeng et al. fabricated Co_2_P/N@Ti_3_C_2_T_x_@NF in which cobalt phosphates were grown on MXene (Ti_3_C_2_T_x_)-modified Ni foam (NF) in a controlled manner by a two-step electrodeposition process. It exhibited an ultralow overpotential of 15 mV at a current density of 10 mA cm^−2^ [164]. Xu et al. prepared a hierarchical shell on the superficial layer of the metal–organic frameworks (MOFs) using interconnected and vertical Ni–Fe-Ce-LDH nanosheets. It showed an excellent OER performance at a current density of 10 mA cm^−2^, exhibiting a low overpotential of 242 mV, and at least 24 h durability was shown [165].

*Electrochemical Nitrogen Reduction (ENR)* Ammonia is greatly significant to industries and humankind in general. Global NH_3_ production got as far as 146 million tons in 2015, and by the year 2050, it is likely to increase by 40% [166]. Over 80% of NH_3_ total production is used for fertilizers which help sustain lives worldwide [167]. For most industrial chemicals, NH_3_ is used as primary raw material, for example, in pharmaceuticals, plastics, dyes, explosives, resins, and synthetic fibers. Also, it has a high energy density and high hydrogen content of 5.52 kWh kg^−1^ and 17.6 wt%, respectively, making it an excellent carbon-free energy carrier [111]. It can be liquified with ease for transportation and storage and is considered an alternative fuel for H_2_ storage.

However, the current ammonia synthesis still depends on the energy-intensive Haber–Bosch process, which needs a high temperature of ~ 300–500 °C and a high pressure of 150–200 atm. With massive amounts of natural gas consumption and emission of CO_2_. Therefore, developing an approach that is green and sustainable for NH_3_ synthesis is quite critical. For their growth, many plants convert atmospheric N_2_ into NH_3_ fertilizer by an enzyme called nitrogenase. However, due to the ultralow reaction rate of nitrogenase in the artificial N_2_ fixation process, research is aimed toward the development of other approaches which can be more advantageous. Catalytic synthesis of NH_3_ can be conducted under ambient conditions. Hence, photocatalytic and electrocatalytic N_2_ reduction reactions are more effective methods [168].

*Electrochemical Nitrogen Reduction (ENR)* appears to be a promising research area over the last few years [111]. It is an energy-efficient alternative as it can be driven by electricity from renewable energy sources like solar and wind energy. Nevertheless, ENR suffers from some significant challenges, such as the inert nature of N≡N, the low solubility of N_2_ in water, and competing hydrogen evolution reactions. Designing effective electrocatalysts that show a desirable electrocatalytic performance reduces the high activation barrier of the N≡N bond and shows selectivity towards NRR, decreasing the competing HER is challenging. Various catalysts based on 2D transition metals and their oxides, nitrides, and carbides have been proposed for electrochemical NRR. But the exhibition of low overpotential, high Faradaic efficiency, and long stability and durability is still beyond reach. Zhang et al. reported electrocatalyst Fe doped SnO_2_ (Fe-SnO_2_) for ambient NRR, which exhibited a remarkable yield of 82.7 mg h^−1^ mg_cat._^−1^ and faradaic efficiency (FE) of 20.4% for NRR using the acid electrolyte. Apart from this, it served as an excellent electrocatalyst for NOR, showing a NO_3_^−^ yield of 42.9 mg h^−1^ mg_cat_^−1^ and 0.84% FE [169]. Guo reported that MXene/TiFeO_x_-700 nanosheets with modified surfaces showed an excellent FE of 25.44% and 21.9 µg mg_cat_^−1^ h^−1^ NH_3_ yield. This enhanced performance was due to its high surface reactivity obtained by removing inactive F*/OH* terminals and adding Fe to reduce the work function [170]. Peng showed that carbon nitride with N-defects coated on C paper, synthesized at 600 ºC (CN/C600), demonstrates promising nitrogen reduction activity. It achieved an NH_3_ yield of 1.7 µg mg_cat_^−1^ h^−1^ with a high FE of 62.1% at − 0.1 V vs RHE because of abundant vacancies [171].

**Fig. 8:**
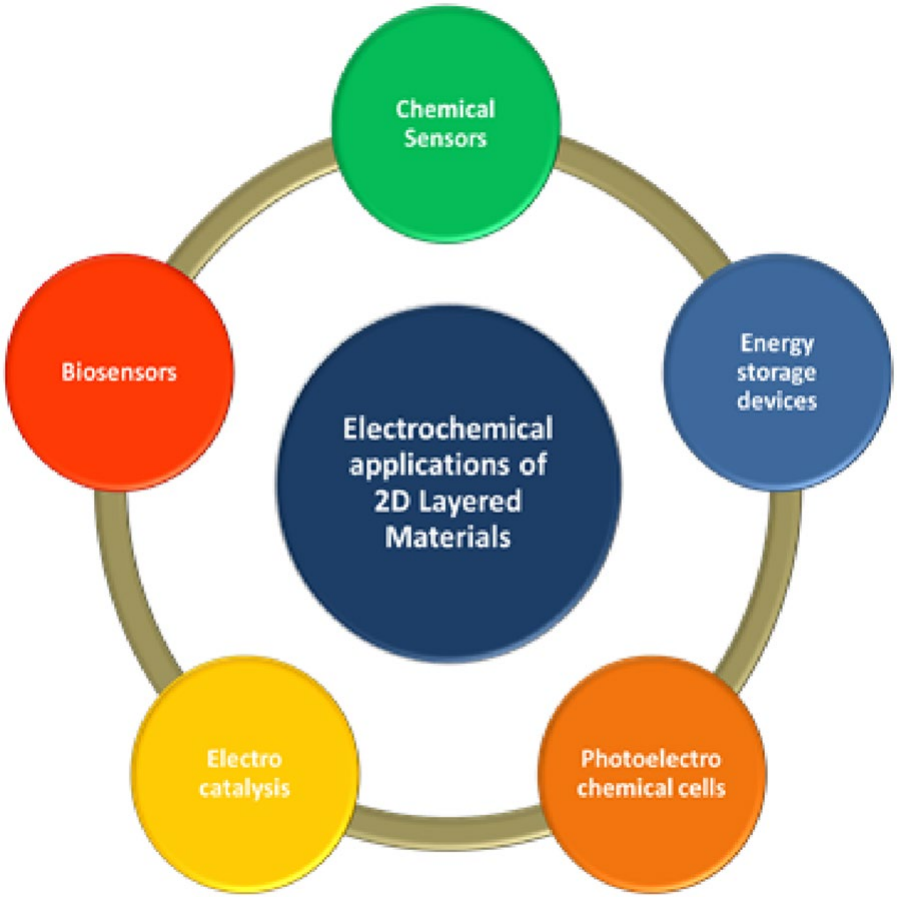
Schematic overview of electrochemical applications of 2D-layered materials

*CO*_*2*_* reduction reaction (CO*_*2*_*RR):* Mesoporous SnO_2_ nanosheets (mp-SnO_2_) reported by Han et al. when evaluated as CO_2_RR electrocatalyst in 0.5 M NaHCO_3_ exhibited a high FE of 83% at − 0.90 V and great stability for production of formate [172]. Yuan et al. reported that 2D amorphous SnO_x_ from liquid Sn-Bi alloy containing fine nanoparticles and single atoms of Bi shows high formic acid FE (> 90%) in CO_2_RR. It also displayed a stable performance over 10 h [173]. Jing Li et al. showed that SnO_2_ nanosheet in a traditional H-type electrochemical cell exhibited a FE > 80% over a large potential window for formate production in CO_2_RR. A three-compartment microfluidic flow cell electrolyzer can achieve 94.2% high FE and 471 mA cm^−2^ current density (Fig. 8) [174].

### Nanofluidics

Nanofluidics easily find applications in electrochemical energy conversion and storage, biosensing, and water purification. The field of 2D nanofluidics is receiving growing interest due to its high versatility, high scalability, and precise and tunable channel size with different types of 2D nanomaterials. The 2D membranes can be used smartly to control the fluid flow through the nanofluidic channels by switching them completely on and off with high efficiency. Fluids such as electrolytes show drastically different flow properties when confined in nanochannels. Two dimensional (2D) nanosheets dispersed in solution on filtration result in a lamellar structured membrane after restacking and drying. The stacked sheets spontaneously form inter-connected nanochannels of sub-nanometer to a few nanometers size which is also comparable to the size of hydrated ions. The 2D layered membrane needs to have high, stable, and modulable ionic conductivity [175]. The ion transport through the membrane is largely dependent on the surface properties of the nanochannels, providing a broad range of properties [176]. In 2D nanochannels when the gap/spacing is shorter than the Debye length of the electrolyte, the surface charges on the walls repel ions of the same charge and attract the oppositely charged ions, making them the dominating charge carriers in contrast to bulk solution, where ions move in opposite directions generating ionic current. Such ionic transport phenomena can increase ionic conductivity up to several magnitudes. A two-order enhancement in ionic conductivity (1 Scm^−1^) of alkali metal ions in two-dimensional MoS_2_ nanosheets is observed in contrast with traditional strong ionic materials [187]. Further, MoS_2_ membrane-based nanofluidic device in which ionic current is enhanced with light intensity is shown in Fig. 7h, i [109]. It is demonstrated in the literature that the electrical properties of 2D materials, the mass transport can be switched between ultrafast to complete blocking [177], and the ion diffusion can be modulated with low voltage [178]. The functionalized MoS_2_ in ultralow concentration can act as a nanofluid decreasing the interfacial tension, changing the contact angle from 131.2° to 51.7°, and imparting emulsion stability by adsorbing at oil/water interfaces [179]. Further, the direction of ion transport and ionic current rectification effect can be reversed with hybrid 2D materials with different surface charges. This includes reconstructing 2D layered materials by asymmetric structure by changing the order in which layers are deposited over one another. For example, the preferential direction for ion transport is revered by altering the WSe_2_ and MoS_2_ layers. The ionic current rectification effect in 2D nanofluidic heterostructures provides further opportunities for innovative nanofluidic devices and materials [180]. Free-standing Boron Nitride membranes can be used to fabricate parallel slit-shaped ionic nanochannels with a highly negative zeta potential of − 33.9 ± 0.9 mV (pH ∼ 7) [181]. LDH (Layered Hydroxide) coating membrane with a conductive polymer allows high ionic conductivity and enhanced selectivity through nanochannels [182]. Polydopamine with excellent ion affinity is coated on the surface of 2 D layers to form composite conductive materials with enhanced electric and ionic conductivity [183]. There are many problems associated with the control of fluid flow through nanochannels such as complex preparation of membrane and the lack of charge density stability in the aqueous medium. The composite membrane of cellulose nanofiber with MXene nanosheets shows high tensile strength (171.2 MPa) and aqueous stability and is used for the fabrication of elevated osmotic conversion devices [184]. In literature, various efforts have been made to control the orientation of nanochannels. For example, Zirconium—Graphene Oxide composite is designed with Z-directional transport of ions for improving the thermal stability and molecular selectivity of graphene oxide [185]. Conventional nanofluidic sieving devices fabricated with three-dimensional nanostructures restrict the molecules to ~ 20 nm, while two-dimensional materials offer a sieving resolution of ~ 0.6 nm, which is remarkable. A nanofluidic sieving device is fabricated using hexagonal boron nitride and in-plane graphene recently with an ultrahigh-resolution to separate a single nucleotide [186]. A further implication of nanofluidics in particle transport applications requires 2D materials with enhanced ionic conductivity, stability in different environmental conditions along with patternable and adaptability for miniaturization.

### 2D material as memory devices

An increase in the demand for storage capacity and miniaturization of the devices has fueled the research on low dimensional materials for the realization of the von Neumann architecture having independent logic and memory devices. In the recent past, Complementary Metal Oxide Semiconductor is also known as CMOS technology (and is the most accepted technology for design, integration, and manufacturing of the integrated circuits) based on the conventional silicon field-effect transistor (FET) [188] fails to deliver at nanometer technology. In addition, conventional memory devices such as SRAM (Static Random-Access Memory), DRAM (Dynamic RAM), and flash memory are also facing crucial challenges such as power dissipation, robustness, reliability, time-based performance degradation, low on/off ratio, etc. [189–192]. Therefore, Ferroelectric Field Effect Transistors (Fe-FETs), Magnetic Random Access Memory (MRAM), Resistive RAM (RRAM), Phase-Change RAM (PCRAM), and Mem-resistor have been significantly engineered and developed as plausible non-volatile memory device technologies. Interestingly, IBM and 3D-Xpoint have developed and designed memory devices based on spintronics, which is currently being envisaged as an alternative to MRAM [193–195]. However, the challenges such as cost, efficiency, shelf-life, speed, and on/off ratio still need significant improvisation to realize these memory-based upcoming devices. 2D materials having achievable electrostatic gate control such as graphene, transition metal dichalcogenides, phosphorene, and other heterostructures as a semiconducting channel have shown promising results in outcome Si-based FETs [196–209] and with unique intrinsic properties (magnetic moment/electric dipoles) for flash memory devices.

2D material-based flash memory was first reported by Zhan et al. [188] by using graphene (a monoatomic thick sheet of the carbon atom, arranged in a hexagonal pattern) on a silicon/silicon oxide (Si/SiO_2_) layer on a substrate having hafnium oxide on top of the graphene sheet and nickel nanoparticles embedded in the between two layers of hafnium oxide. It was observed that the on/off ratio of the device was poor and the consumption of energy is significantly high as compared to other flash memory devices. This experiment attests to the capacity of the 2D materials as flash memory and opened a new domain of research in the field of layered materials. Later in 2013, Yang et al. investigated the flash memories in memristors and reported that they have a memory retention time of 10 years and have an ultra-fast writing speed of ~ 10 ns. [189] Moreover, with the invention of transition metal dichalcogenide having a bandgap of the order of ~ 1.4 to ~ 1.9 eV (depending upon thickness), a graphene and molybdenum disulfide floating gate flash memory device was realized by Gurarsalan et al. [190]. It was observed that the on/off ratio and energy consumption was dramatically reduced in comparison to the report by Zhan et al. Moreover, many researchers such as Zhang et al. [191], Li et al. [192], and Feng et al. [193] have worked on a similar kind of floating gate TMDCs device using the structure as MoS_2_/Al_2_O_3_/HfO_2_/Al_2_O_3_, BP/h-BN/MoS_2_, and BP/Al_2_O_3_/HfO_2_/Al_2_O_3_ respectively. Although flash memories are well established for layered 2D materials, power consumption for the frequent refresh was the most challenging problem being faced and needs to be overcome. Semi-floating gate memory devices were seen as one of the alternatives to flash memory-based devices and soon made their inception in 2018 by Liu et al. [194]. It was the first report on the quasi-non-volatile memory devices and opened a new paradigm of applications using 2D materials. Liu et al. demonstrated the device performance by using tungsten selenide (WSe_2_) /molybdenum disulfide (MoS_2_) /boron nitride (h-BN)/hafnium oxide (HfO_2_) structure. The heterostructure structure was designed such that the charges from the WSe_2_-MoS_2_ layer formed a2D p–n junction thus transportation of charges can be made swiftly. In addition, the role of the HfO_2_ layer was to limit the transfer of charge from the floating gate thus decreasing the power consumption and increasing the operation speed. Although the writing speed was enhanced in the single floating gate devices, the erase speed was now a challenge to overcome. Li et al. in 2019, [195] overcame this challenge by introducing 2D material p–n junction floating gate of WSe_2_-MoS_2_.

Before discussing the FETs that have circuit complexity and usually have high power consumption due to the presence of the oxide layer in comparison to two terminal floating devices. It would be advised to know about the two-terminal memory devices. In FET, the presence of the oxide layer further restricts the growth in the Z direction (vertically) and limits the flexible nature of the devices [196–210]. However, the two-terminal devices have extremely short channel lengths and the absence of a gate electrode makes them a promising memory device. The two-terminal devices are further classified into two types, namely, Phase-change random-access memory (PRAM) and resistive random-access memory (RRAM) [211–213]. In comparison to FETs, the two-terminal devices have a low on/off ratio and off-state power consumption.

Vu et al. [214] 2016 reported a tunneling random access memory device and have demonstrated the performance of the TRAM in comparison with FETs. In addition, Lee et al. [215, 216] designed a heterolayer graphene/h-BN/MoS_2_ much similar to that presented by Choi et al. [217]. The seminal report by Lee et al. has used drain field-induced tunneling charge carriers to write and erase. Moreover, the two-terminal device has laid the stepping stone for layered material-based TRAM and was eventually realized that the TRAM has better efficiency, performance, and potential for the upcoming flexible electronics [218–220].

2D materials have been realized as a potential floating gate material and a variety of them have been used as a non-volatile memory or as flash memory to improve the figure of merit of the devices. Graphene as flash memory helps in reducing the gate stack and minimizing the capacitive coupling. Following the success of graphene in the non-volatile memory, most of its cousins such as transition metal dichalcogenides (MoS_2_), boron nitride, black phosphorus, etc. have been investigated and thus created a limited 2D material based non-volatile memory devices and their performance in respect to graphene and conventional silicon have been compared and summarized in Table 1.

**Table 1:** 2D material-based non-volatile memory devices and their performance

Active layer (thickness)/year	Current switching ratio	Set voltage [V]	Retention	Endurance in cycles	Ref./Year
Graphite [2008]	1.5 × 107	4–6	2 weeks	> 103	[209]
Graphene (1–2 L) [2008]	102	~ 6	24 h	> 105	[210]
Graphitic stripes (~ 10 nm) [2009]	107	3–4	–	2.2 × 104	[211]
GO thin film (~ 30 nm) [2009]	20	0.3–1	104 s	> 100	[212]
GO thin film (~ 15 nm) [2010]	103	2.5	105 s	> 100	[213]
GO (~ 30 nm) [2010]	103	1.6	107 s	100	[214]
GO thin film (50–100 nm) [2011]	103	0.7	–	–	[215]
RGO thin film (20 nm) [2011]	105	7.5	103 s	> 100	[216]
Graphene (1 L) [2012]	106	7	104 s	–	[217]
rGO-ferrocene film (~ 50 nm) [2012]	103	2	103 s	> 103	[218]
RGO-Au [2011]	2	5	103 s	> 20	[219]
MoS_2_–PVP (70 nm) [2012]	102	3.5	–	–	[220]
PtAg–MoS_2_ nanobelts in PVP [2014]	–	5	DRAM	–	[221]
MoS_2_/GO hybrid film (~ 100 nm) [2013]	102	1.2	–	–	[222]
GO/MoS_2_/GO (total ~ 20 nm) [2016]	104	4	104	102	[223]
MoS_2_–PMMA [2017]	102–103	2	105	105	[224]
MoS_2_–PVA [2016]	102	3	104	103	[225]
MoOx/MoS_2_ (50–600 nm) [2015]	106	0.1–0.2	104	104	[226]
1 T-MoS_2_ film (~ 550 nm) [2016]	103	0.1	–	103	[227]
CVD MoS_2_ (~ 1 L) [2015]	103	8.3	120	–	[228]
CVD h-BN (~ 3 nm) [2016]	102	0.72	3 × 103	550	[229]
CVD h-BN (5–7 L) [2017]	10	0.4	–	> 350	[230]
BP (~ 3 µm) [2015]	3 × 105	1.5–2	105	–	[231]
p-type Si (bulk) [2010]	7.5	–	–	–	[232]
p-type Si (bulk) [2011]	16.8	–	10 years	–	[233]
Graphene (1 L) [2013]	20	2	1200 s	> 100	[234]

### Field emission in 2D materials

Field Emission is one of the most demanding upcoming technologies in the field of electronics (flat panel displays, electron microscopy, traveling wave tubes, etc.) driven and governed by quantum tunneling. It involves exposure of materials to high external fields and ultra-high vacuum conditions for the emission of electrons. The three principles of working parameters that define the field emission process are (a) semi-classical thermionic emission, (b) quantum mechanical tunneling, and (c) photoemission. Out of the mentioned technique, thermionic emission is a thermally driven process that incorporates thermal energy which often leads to the emission of surface electrons and thus relies on the Joule-heating method [234]. However, in general, in the field emission process, an electron forms a semiconductor surface tunnels through the vacuum under influence of a high external electrostatic field and ultra-high vacuum at the standard room temperature and pressure [235, 236]. This process is called quantum mechanical tunneling and is also recognized as cold emission at room temperature (usually occurs due to the vacant states available at the Fermi level and the applied electric field). Moreover, in photo-emission, the electrons overcome the potential barrier height by absorbing the incident energy source. In the case of photo-emission, the electrons energetically overcome the potential barrier by gaining enough energy from the incident photons. Thus, the incident photon must be energy higher than the work function (in the case of solids) and/or binding energy of ejecting electron [237–239].

**Fig. 9:**
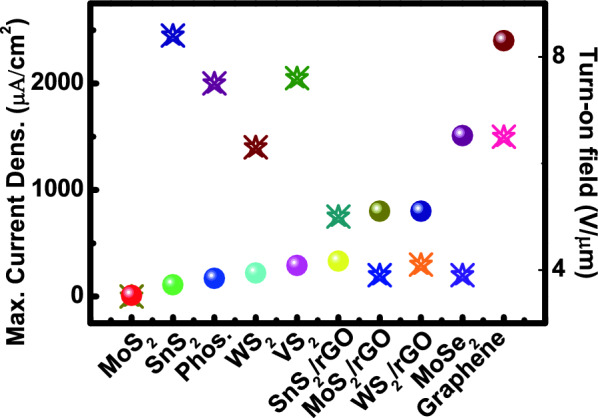
Field Emission parameters of the 2D materials and their hybrids

Graphene and its cousin such as molybdenum/tungsten/tin/vanadium sulfide/selenide/disulfide, and phosphorene, as well as their hybrids often offers high field enhancement from the edges and localized defect sites due to the low dimensionality and quantum confinement and is thus being pursued with great scientific interest. It was found that the turn-on field for graphene, WS_2_, VS_2_, SnS_2_, MoSe_2_, Black phosphorus, ZnO sheets, MoS_2_-SnO_2_, MoS_2_-MWCNT, WS_2_-rGO, SnS_2_-rGO, MoS_2_-rGO are 3.5, 2.3, 4.6, 5.01, 7.5, 2.3, 4.2, 2.4, 3.4, 2.7, 2.0, 2.65 and 2.6 V/µm respectively (see Fig. 9) [240–252].

However, 2D materials are yet to be used as cold cathodes and yet to be investigated based on the parameters or their performance which include but are not limited to lower turn-on electric field (E_on_), lesser threshold electric field (E_thers_), higher maximum current density, higher field emission factor and high current stability with fewer fluctuations. In addition, some seminal reports suggest that these 2D materials are further tuned and investigated by different techniques such as doping, inducing defects, creating vacancies, surface charge tuning, the inclusion of gate voltage, tuning the thickness, inducing thermal effect, hybridization and composites.

## Future generation devices and sensors

**Fig. 10:**
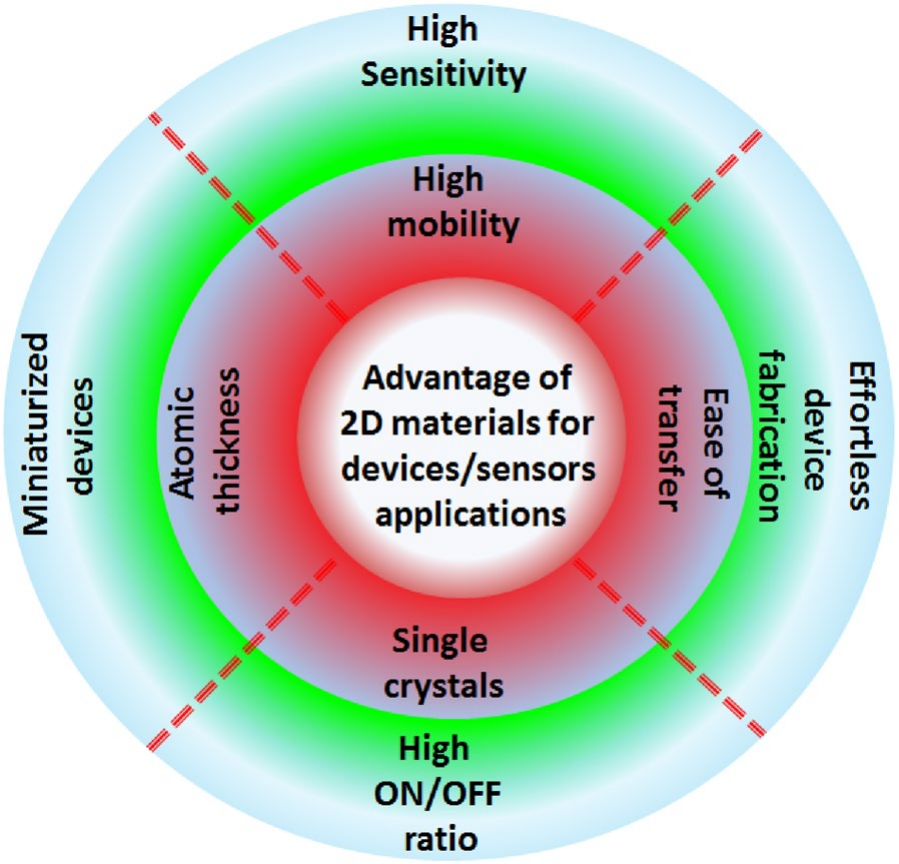
Salient advantages of 2D materials for device/sensing applications

The advent of 2D materials has completely transformed the face of material science and engineering. It also paves the way to approach Moore’s law which has otherwise been slowed down due to technological limitations and fueled the fabrication of next-generation semiconductor devices. In addition, the interesting attributes offered by the members of the 2D materials family and the low power consumption in particular; further augments its frontline applications where nanocrystalline thin films and nanowires miserably failed. It should be noted that the past 30–40 years have seen enormous applications of these nanomaterials of compromised dimensions and off late, it has hit the walls of limitations. 2D materials provide further breathing space and it seems that it has filled the technological gap. Some of the unique characteristics of 2D materials based on the van der Waals and non-van der Waals structure have been explicitly discussed in the article and to summarize the advantage of these advanced materials vis-à-vis their bulk counterpart, numerous scientific data published by leading scientists and researchers, have been collected from the literature and represented in Fig. 10. It is evident from the figure and the discussion in the article that even though graphene in its pure form being a semi-metal has excellent electronic mobility, yet could not exhibit the high ON/OFF ratio, due to lack of carrier concentration and lack of bandgap, other 2D semiconducting cousins do exhibit high ON/OFF ratio. Moreover, doping of graphene and graphene-based hybrids do have the potential for electronic chip applications. Apart from the high carrier mobility (necessary for fast sensing), thermal conductivity (apt to remove extra heat which hampers swift carrier transport), high Young’s moduli (good for flexural behavior), and optically transparent nature, these advanced quantum 2D materials have been employed in ultrafast gas/molecular/light/fire/strain sensors, microwave shielding, Flextronics, in hybrid energy storage and mechanically strengthening polymers (see Fig. 10).

**Fig. 11:**
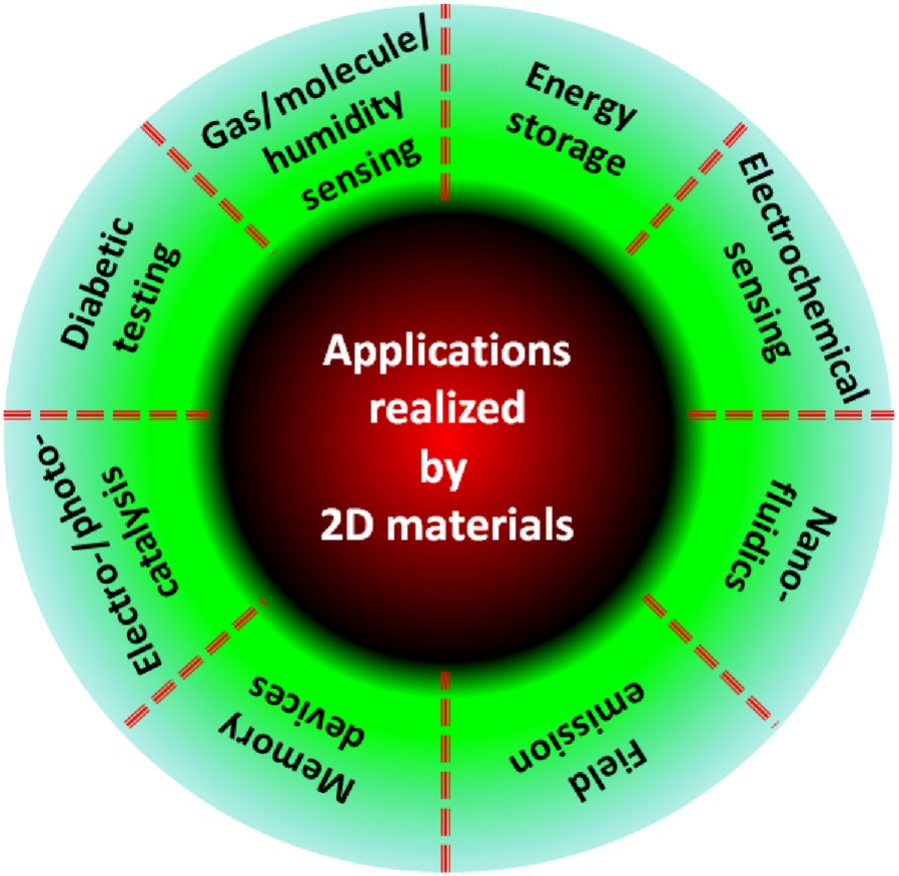
Applications are already realized by 2D materials, doped 2D materials, and their hetero layers

2D materials being atomic thick, have unprecedented physical and chemical properties which are better than their bulk counterpart. We have discussed the role of 2D materials from light emission (field emission), and analyte detection to energy generation and storage (Fig. 11). However, the properties shown by the 2D materials can be further enhanced by doping, hybridization, intercalation, and by heterostructure design. In addition, the article also summarizes the synthesis of various 2D materials and their characterization. It should also be mentioned that these 2D materials although seem to be easy to synthesize, it is difficult to make devices out of them due to certain challenges as discussed in Fig. 14. Amongst various challenges toxicity is one of the issues which need to be addressed. Apart from graphene which originates from carbon, other 2D materials such as alpha lead oxide, phosphorene, and TMDCs are mildly toxic and therefore appropriate precautions in handling are desirable. The other major challenges with these 2D materials during the fabrication of transistors are the lack of current saturation and low current gain. In addition, the pin-hole-free layered devices are free from defects and are suitable to grow on the arbitrary substrate (minimizing mismatch between substrate and film), reusability of the film and substrate, avoiding contact resistance, scalability in synthesis, and larger crystal grain are some of the upcoming challenges which further need to be addressed.

**Fig. 12:**
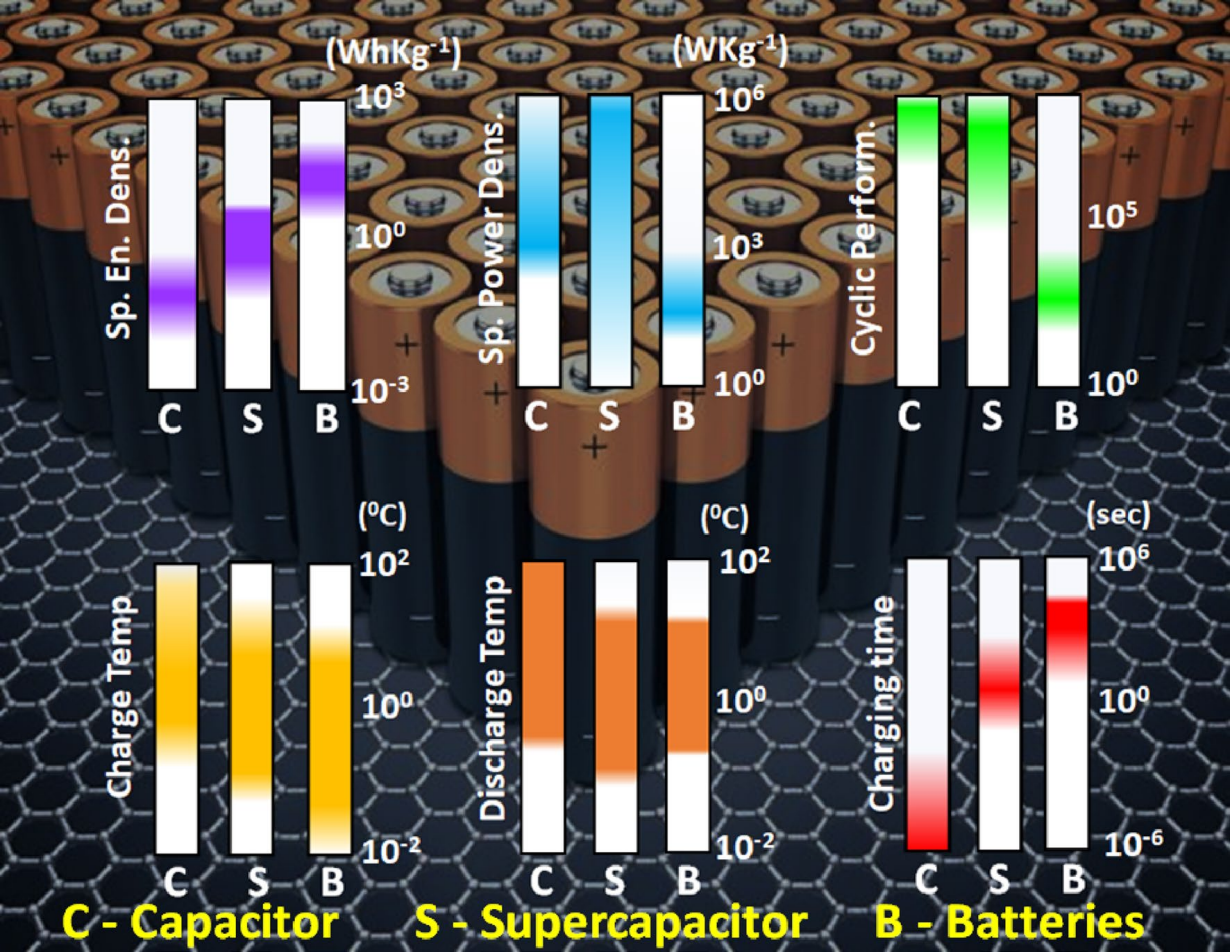
Summary of critical parameters deciding capacitor, supercapacitor and batteries performance

In general, 2D materials has high surface area, high mobility (electron and ionic mobility respectively) high thermal conductivity, structural integrity (especially in borophene and other 2D materials). Some of the 2D materials have atomic scale channels due to ridge line formation in its lattice structure for example borophene, phosphorene, TMDCs etc. which eventually helps in directional electronic or ionic mobility. However, the two critical parameter which generally decide performance and stability of the device (energy storage and generation) are electronic structure and thermal stability. It was found that the specific energy density of the capacitor, supercapacitor and batteries should lie in the range of 0.01–0.1, 0.1–50, 10–200 Wh Kg^−1^ (as shown in the Fig. 12)_._ In addition, the specific power density, should be 10^3^ to 10^7^, 1–10^6^ and 10–100 (W Kg^−1^) respectively, cycling performance be like infinite, > 500,000 and 500 ~ 2000 respectively and charging time in sec be 10^–6^ to 10^–3^, 1–10, 10^3^ to 10^5^ respectively for capacitor, supercapacitor and batteries (as shown in the Fig. 12).

**Fig. 13:**
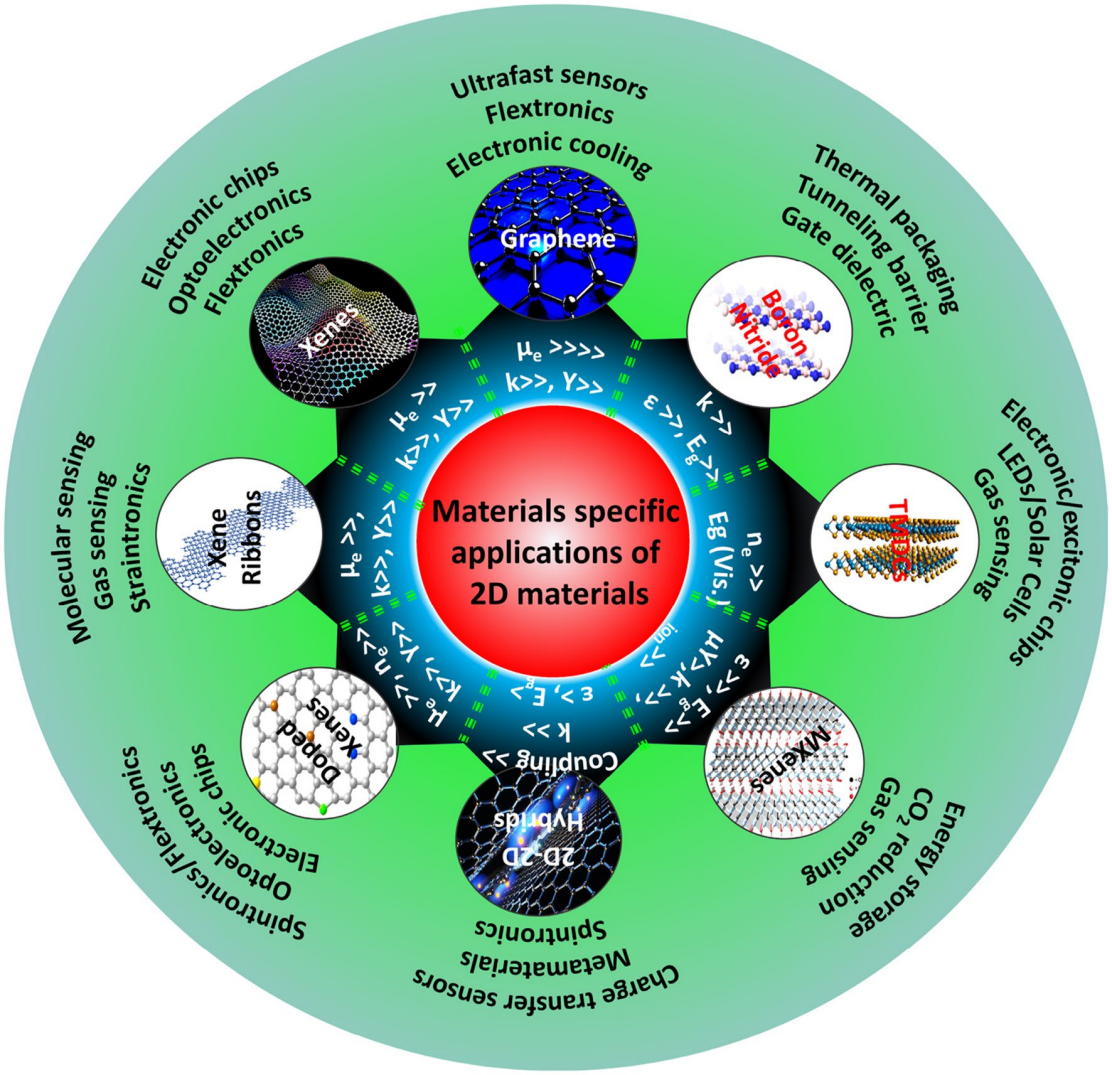
Materials specific applications of various classes of 2D materials

Numerous applications, challenges and properties of 2D materials need to be investigated before realizing end usage application in terms of devices. A broad perspective to understand and realize these crucial parameter (namely electronic mobility, ionic mobility, young’s modulus, thermal conductivity) along with the suitable applications in terms of device performance have been summarized (see Fig. 13). It is evident from the Fig. 13 that the highest electronic mobility can be realized for a device is from graphene. However, it seems that it lags bandgap and thus the ON/OFF ratio is poor which eventually obsolete its use in FETs unless doping or hybridization is being considered with other 2D/3D materials. The ON/OFF ratio of the electronic chip depends on the bandgap of the material and thus other 2D material are being explored with great scientific interest. In light of the ON/OFF ratio it was found that anisotropic materials especially borophene is a promising material for FETs. Xenes ribbons are similarly being investigated for straintronics, gas sensing, and molecule sensing. However, when doped with different atoms its being an interesting material for spintronics, optoelectronics etc. Boron nitride the best insulator being found in the 2D family is currently being a sensation for application in thermal packaging, tunneling barrier and gate dielectric due to high thermal conductivity. TMDCs are apt for band gap engineering based optoelectric applications such as in LEDs and solar cells. MXenes do possess stable structure and band gap adequate for energy storage and water splitting and catalytic CO_2_ conversion.

## Primary challenges

Obtaining defect-free atomically thin crystal is in general difficult. Growing or transferring such crystal on arbitrary substrate is a challenge in itself. Many approaches of synthesis (Bottom-up methods such as MPCVD and laser CVD; Top-down methods such as laser exfoliation, direct exfoliation using PDMS stamp etc.) and adequate lithography (e-beam, ion-beam and laser beam, AFM-tip based) are therefore being developed to address to these challenges. To have atom-by-atom information, high resolution HAADF HRTEM imaging, atomic-scale elemental profiling via STEM EELS is being employed. XPS and Raman mapping have come very far to diagnose the specific surfaces such as doped Xenes, where local information is desirable. In fact, uniform graphitic doping is desirable, however not very straight forward. Therefore, new ways to attain such uniformity in doping are being developed. Further, new approaches to accomplish strong 2D-2D hybridized materials are being explored. In general, for 2D-2D hybrids, getting interfacial information is difficult. HRTEM, Raman mapping etc. can help diagnose the effect, however exact interpretation is difficult as there are many factors such as Moire potential developed (due to shear, translation, rotation of crystals relative to each other), unwanted stacking, voids/trapped air at the interfaces etc. All these factors give rise to undesirable noises in electrical signal. In fact, room temperature electrical signal is very noisy. Therefore, room-temperature diagnosis tools such as scanning tunneling microscopy (STM) or current force microscopy (CAFM) based electrical signals for evaluating the quality of interfaces are being developed [253].

**Fig. 14:**
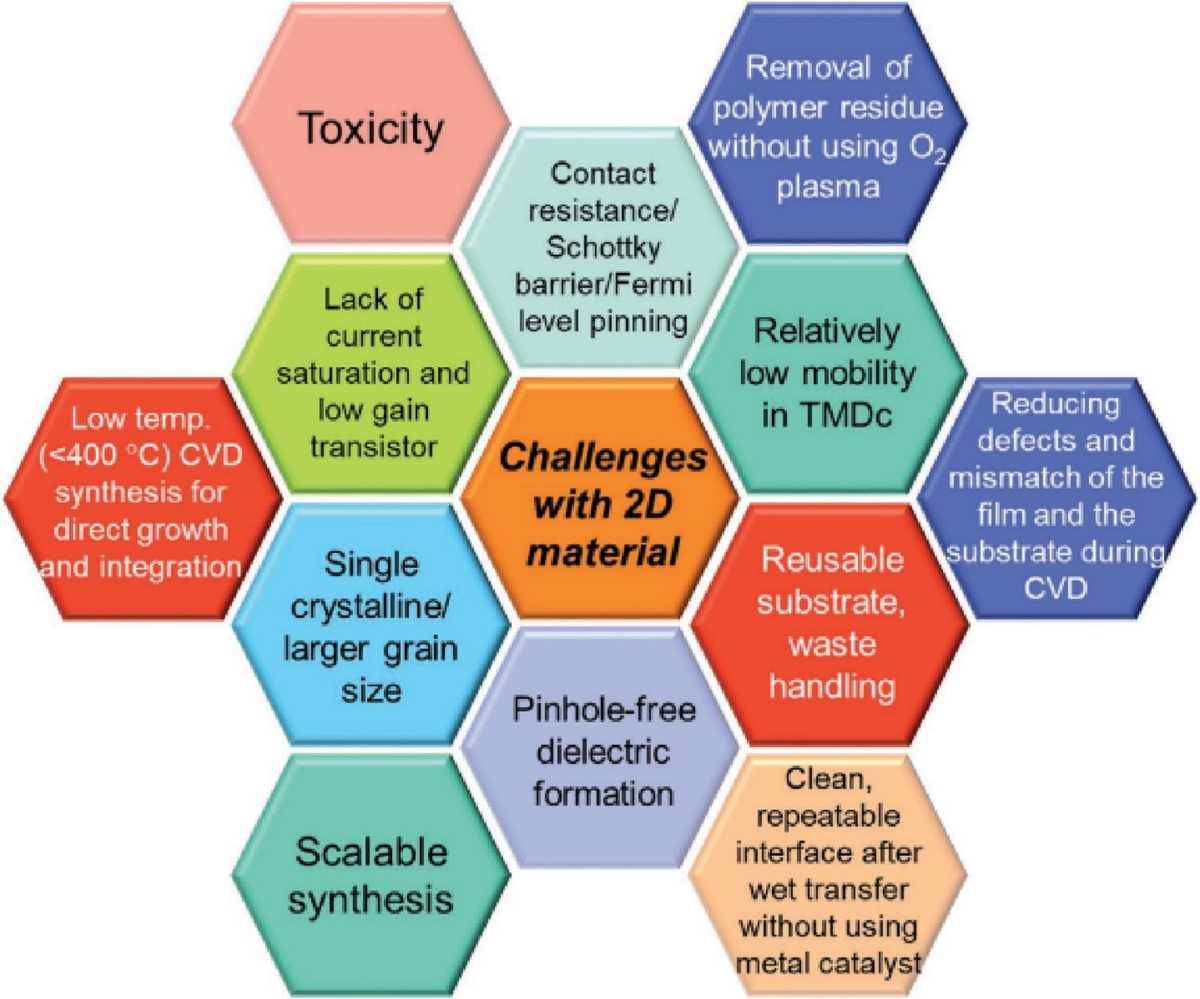
Challenges faced by 2D materials from synthesis to device applications [50]

The 2D materials for electrocatalysis need defect engineering to improve catalytic performance and increase the number of active sites and the electronic conductivity of the material. A fundamental understanding of the electrocatalytic behavior of a material and its correlation with defects require further enhancement. The development of multi-functional electrocatalysts is needed by rational defects design and other routes for a clean, sustainable, and low-cost energy conversion [254]. 2D-layered electrode materials in supercapacitors provide excellent areal/volumetric capacitance with high energy density, power density, and long life cycles at a relatively low cost. The synthesis of a single 2D layer requires multiple-step processes and involves both considerable time and money. The production of high-quality 2D layered material at an industrial scale is a big challenge because of the high chemical activity and storage of 2D materials. The performance of 2D materials is hindered by atmospheric factors like humidity, electrolyte evaporation due to high temperature, and undesirable chemical reactions. The nanocomposites of 2D layered materials with other conducting materials may solve many of these issues with the cost of some drop in performance but are applicable for practical applications in electrocatalysis [255]. In nanofluidics, 2D materials often undergo reduction or cross-linkage instead of forming a desirable laminar structure. In addition, the electrostatic repulsion between nanosheets membrane can disintegrate in the aqueous medium. Moreover, the cross-linkage can change the hydrophilicity, creating undesirable changes in fluid flow [174]. The cost-effective device manufacturing technologies need to be addressed for flexible, miniaturized, and industrial-scale gas sensors [256, 257]. The synthetic strategies which provide reasonable control over the layer thickness, defects, and composition needs to be developed to enhance the selectivity and sensitivity for optimum performance [88]. One of the significant issues with device fabrication is to make ohmic contact between 2D nanomaterials and metal contacts, as it requires sophisticated techniques and skilled labor, which indirectly sums up to increase the device cost [91]. Generic challenges in working with 2D materials are shown in Fig. 14.

**Fig. 15:**
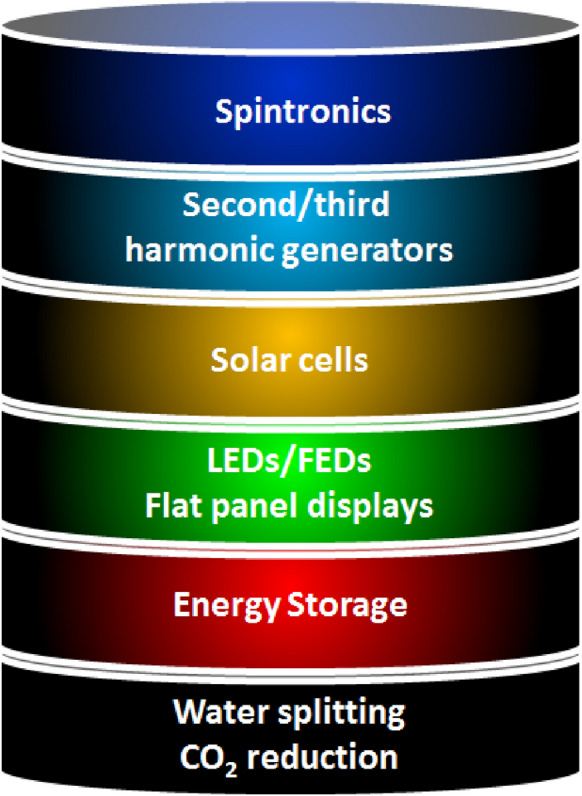
Futuristic applications of 2D materials, doped 2D materials, and their hetero layers

Although 2D materials based devices are facing device fabrication challenges due to scalability issues, especially device integration issues, interconnects etc., with new technologies and better understanding of these material properties they are highly sought after materials for supercapacitors due to high surface area and ionic conductivity, magnetic devices as single doped atoms/functionalities on same lattice and different lattices can induce spin oriented magnetism also called as spintronics, flexible displays due to flexural behavior and also due to optical transparency and tunable electronic bandgap, energy storage due to high specific surface area, water splitting due to control over the kinetics of the material domains, filtration by inducing atomistic level defects which eventually will obsolete analytes such as sodium, potassium etc. and prevent fouling as well as maintains the Young’s modulus, lubricants by decreasing the friction due to presence of weak van der Waals force of attraction, second/third harmonic generators, dye adsorbents due to control over surface charge density and distribution etc. (see Fig. 15). Bioelectronics and future lab-on-chip devices based on 2D materials is yet another applications, 2D materials is catching up in recent times [258]. Field emission applications of 2D quantum crystals is extremely interesting from field emission display application point of view [259]. Large scale applications of 2D materials are very promising e.g. in mechanically strengthening glass (for bulletproofs), aluminum (for light combat aircrafts), steel rods (for earthquake resistant residential towers and rust-proof river/ocean bridges), plastic sheets (for various flexural devices/appliances) etc. Thus, we see that within no time, quantum flatland has broadened laterally and has encompassed a myriads of applications.

## Conclusions

The evolution of the family of 2D materials consisting of monoelemental atomic sheets called Xenes, boron nitride, TMDCs, MXenes, etc. has been described and discussed in detail. Important synthesis protocols for these advanced materials have been summarized. Characterizations of 2D materials have been elaborated with an emphasis on chemical identification and electronic character determination. Salient practical applications of 2D materials primarily in energy storage, gas and humidity sensing, electrochemical sensing, electrocatalysis, nanofluidics, memory devices, and field emission have been discussed. At the end of the review, the future generation of devices and sensors have been elaborated and a perspective has been presented.

## Data Availability

Not applicable.

## Abbreviations

FESEM: Field emission scanning electron microscopy
AFM: Atomic force microscope
TEM/HRTEM: Transmission electron microscope/ high-resolution transmission electron microscope
XPS: X-ray photoelectron spectroscopy
FTIR: Fourier transform infrared
FET: Field-effect transistor
LEDs: Light emitting diodes
SERS: Surface enhanced Raman spectroscopy
TMDCs: Transition metal dichalcogenides
HER: Hydrogen evolution reaction
OER: Oxygen evolution reaction
MOFs: Metal oxide frameworks
RH: Relative humidity
VOC: Volatile organic compounds
ENR: Electrochemical nitrogen reduction
ppb: Parts-per-billion
ppm: Parts-per-million
RAM: Random access memory
BN: Boron nitride
BP: Black phosphorus
TMOs: Transition metal oxides
MnO_2_: Manganese dioxide
NiCo_2_O_4_: Nickel cobaltite
RuO_2_: Ruthenium (IV) oxide
Fe_3_O_4_: Iron (II, III) oxide
NiO: Nickle oxide
Co: Cobalt
Cu: Copper
MoS_2_: Molybdenum disulphide
WS_2_: Tungsten disulfide
TiS_2_: Titanium disulfide
NbS_2_: Niobium disulfide
VS_2_: Vanadium sulfide
WS_2_-PANI: Tungsten disulfide/polyaniline
kHz: Kilo-hertz
CNT/GO: Carbon nanotube/graphene oxide
CoFe_2_O_4_: Cobalt ferrite
MWNTs: Multi-walled carbon nanotubes
Au: Gold
PET: Polyethylene terephthalate
NPs: Nano-particles
SnO_2_: Tin oxide
WO_3_: Tungsten trioxide
MoO_3_: Molybdenum trioxide
TiO2: Titanium oxide
α-Fe_2_O_3_: Alpha Iron (III) oxide
NH_3_: Ammonia
rGO: Reduced graphene oxide
nM: Nanomolar
COOH: Carboxylic acid
SnS_2_: Tin selenide
HfO_2_: Hafnium oxide
μWh/cm^3^: Micro-watt-hour per centimetre cube
mF cm^−2^: Milli-farad per centimetre square
A g^−^^1^: Ampere per gram
Fg^−^^1^: Farad per gram
mVs^−^^1^: Milli-volt per second
